# Mechanically Robust Supercrystals from Antisolvent-Induced Assembly of Perovskite Nanocrystals

**DOI:** 10.1021/acsnano.5c07289

**Published:** 2025-07-09

**Authors:** Jonas L. Hiller, Robert Thalwitzer, Ata Bozkurt, Matheus Gomes Ferreira, Richard Hodak, Fabian Strauß, Elke Nadler, Gerard N. Hinsley, Bihan Wang, Kuan Hoon Ngoi, Witold Rudzinski, Ekaterina Kneschaurek, Wojciech Roseker, Michael Sprung, Dmitry Lapkin, Dmitry Baranov, Frank Schreiber, Ivan A. Vartanyants, Marcus Scheele, Ivan A. Zaluzhnyy

**Affiliations:** † Institute for Physical and Theoretical Chemistry, 9188 University of Tübingen, Auf der Morgenstelle 18, 72076 Tübingen, Germany; ‡ Division of Chemical Physics and NanoLund, Department of Chemistry, 5193 Lund University, P.O. Box 124, SE-221 00 Lund, Sweden; ¶ 28332 Deutsches Elektronen-Synchrotron DESY, Notkestraße 85, 22607 Hamburg, Germany; § AGH University of Krakow, al. Adama Mickiewicza 30, 30-059 Kraków, Poland; # Institute of Applied Physics, 9188 University of Tübingen, Auf der Morgenstelle 10, 72076 Tübingen, Germany

**Keywords:** lead halide perovskite, nanocrystals, supercrystals, self-assembly, X-ray diffraction, scanning electron microscopy SEM, atomic force microscopy AFM

## Abstract

Ordered arrays of nanocrystals, called supercrystals, have attracted significant attention owing to the collective quantum effects arising from the coupling between neighboring nanocrystals. In particular, lead halide perovskite nanocrystals are widely used because of the combination of the optical properties and faceted cubic shape, which enables the formation of highly ordered supercrystals. The most frequently used method for the fabrication of perovskite supercrystals is based on the self-assembly of nanocrystals from solution via slow evaporation of the solvent. However, the supercrystals produced with this technique grow in random positions on the substrate. Moreover, they are mechanically soft due to the presence of organic ligands around the individual nanocrystals. Therefore, such supercrystals cannot be easily manipulated with microgrippers, which hinders their use in applications. In this work, we synthesize mechanically robust supercrystals built from cubic lead halide perovskite nanocrystals by a two-layer phase diffusion self-assembly with acetonitrile as the antisolvent. This method yields highly faceted thick supercrystals, which are robust enough to be picked up and relocated by microgrippers. We employed X-ray nanodiffraction together with high-resolution scanning electron microscopy and atomic force microscopy to reveal the structure of CsPbBr_3_, CsPbBr_2_Cl, and CsPbCl_3_ supercrystals assembled using the two-layer phase diffusion technique and explain their unusual mechanical robustness. Our findings are crucial for further experiments and applications in which supercrystals need to be placed in a precise location, for example, between the electrodes in an electro-optical modulator.

## Graphical abstract

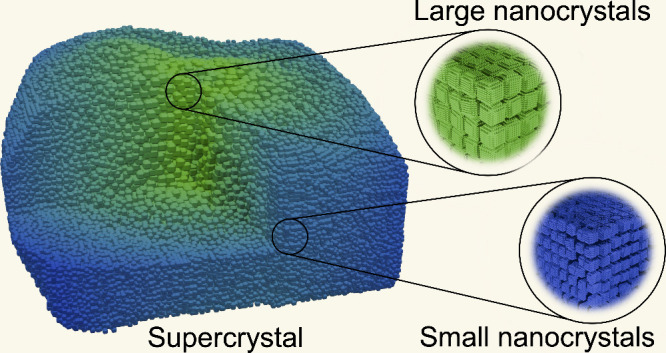

## Introduction

Collective quantum effects, such as superfluorescence, manifest when nearly identical quantum emitters are brought into close proximity and exhibit coupling. Colloidal CsPbX_3_ (X = Cl, Br, I) nanocrystals (NCs) with <10% size distribution have recently emerged as a promising model system in this regard.^1,2^ To further narrow their size distribution and achieve the required uniformity of energy eigenstates,^3^ these building blocks are assembled into ordered arrays referred to as “supercrystals” (SCs), which exploits the purifying effect of colloidal recrystallization.^4–6^ Solvent evaporation and two-layer phase diffusion methods for NC self-assembly have been extensively explored in metallic and semiconductor NCs.^7–12^ Briefly, SCs are preferentially formed by NCs with highly uniform size and shape, while irregularly shaped NCs tend to be excluded from the purified ensemble. Electron microscopy studies of CsPbX_3_ SCs have demonstrated that this effect leads to the selective assembly of NCs with a larger diameter and a narrower size distribution than the ensemble average.^13,14^ The degree of long-range structural order in SCs, particularly the nature and density of defects, has been extensively studied with results ranging from nearly perfect order to considerable structural heterogeneity.^13,15^ Among the many reasons for this variety are different crystallization strategies,^1,16–20^ residual strain during drying of the solvent,^21^ and the softness or density of the NC ligand shell.^22^

Ligands and their softness also play a key role in the mechanical strength of SCs.^23–25^ While structural order in SCs increases the mechanical strength compared to disordered ensembles,^26^ SCs with typical ligand shells, such as oleic acid, are weak with elastic moduli typically less than 1 GPa,^27^ rendering their mechanical manipulation with microgrippers or microtomic loops essentially impossible. Therefore, a number of hardening strategies have been developed for SCs, for example, the polymerization or cross-linking of ligands,^28,29^ O_2_ plasma treatment,^30^ focused ion beam milling,^31^ and exchange with all-inorganic ligands.^32^ With these strategies, elastic moduli have been increased manifold (often reaching values of tens of GPa) that warrant the mechanical manipulation of single crystals, e.g., similar to protein crystals. However, these examples have been limited to relatively inert NCs like Fe_3_O_4_,^28,29^ ZrO_2_,^30^ Au,^31^ CdSe,^32^ or PbS,^33^ while for CsPbX_3_ NC materials, no comparable strategy is available so far.

Here we argue that the pronounced ligand lability of CsPbX_3_ NCs^34^ should provide scope for *in situ* hardening of SCs during their formation with the aid of an antisolvent that reduces the density of the ligand shell.^35,36^ While there has been significant attention given to how antisolvents influence the surface chemistry of individual CsPbX_3_ NCs,^34,37^ there are no studies yet examining how the presence of an antisolvent affects their self-assembly into SCs.

We apply a two-phase (antisolvent/solvent) layer diffusion technique^9,11^ to form CsPbX_3_ SCs, probe their spatially resolved structural properties by X-ray scattering with a focused beam, assess the mechanical properties of the SCs with atomic force measurements, and compare these values with typical results for SCs obtained with the conventional slow evaporation method. We find that CsPbX_3_ SCs grown in the presence of an antisolvent tend to be larger and more faceted. The constituting NCs in all SCs exhibit a broadened size distribution and a gradual increase in the lattice constant from the edge toward the center, which we hypothesize to be due to postsynthetic growth in the presence of the antisolvent. The elastic modulus increases from approximately 0.14 GPa for the SCs fabricated by the solvent evaporation method to 3.2 GPa for the SCs fabricated by the two-phase layer diffusion technique, enabling the mechanical manipulation and relocation of the latter SCs with microgrippers.

This work demonstrates that it is possible to select and isolate individual CsPbX_3_ SCs for optical studies or applications.^38,39^ Furthermore, this suggests that SCs with long-range structural order can tolerate gradients in the size of the constituting NCs, similar to heteroatomic crystals with a graded alloy composition.

## Results and Discussion

We self-assemble SCs from solutions of CsPbX_3_ NCs on a Si substrate with the two-layer phase diffusion technique illustrated in Figure 1a, using hexane and acetonitrile as the solvent and antisolvent, respectively. With this technique, cubic and strongly faceted SCs grow exclusively at the interface between the two phases (Figure 1b) with a typical lateral size of 10 × 10 μm^2^ and a height of 8 μm (Figure S13 in the Supporting Information (SI)). After drying, the SCs are mechanically robust enough to be relocated by microgrippers from the Si substrate on which they were grown to a substrate of choice (Figure 1c).

**Figure 1:**
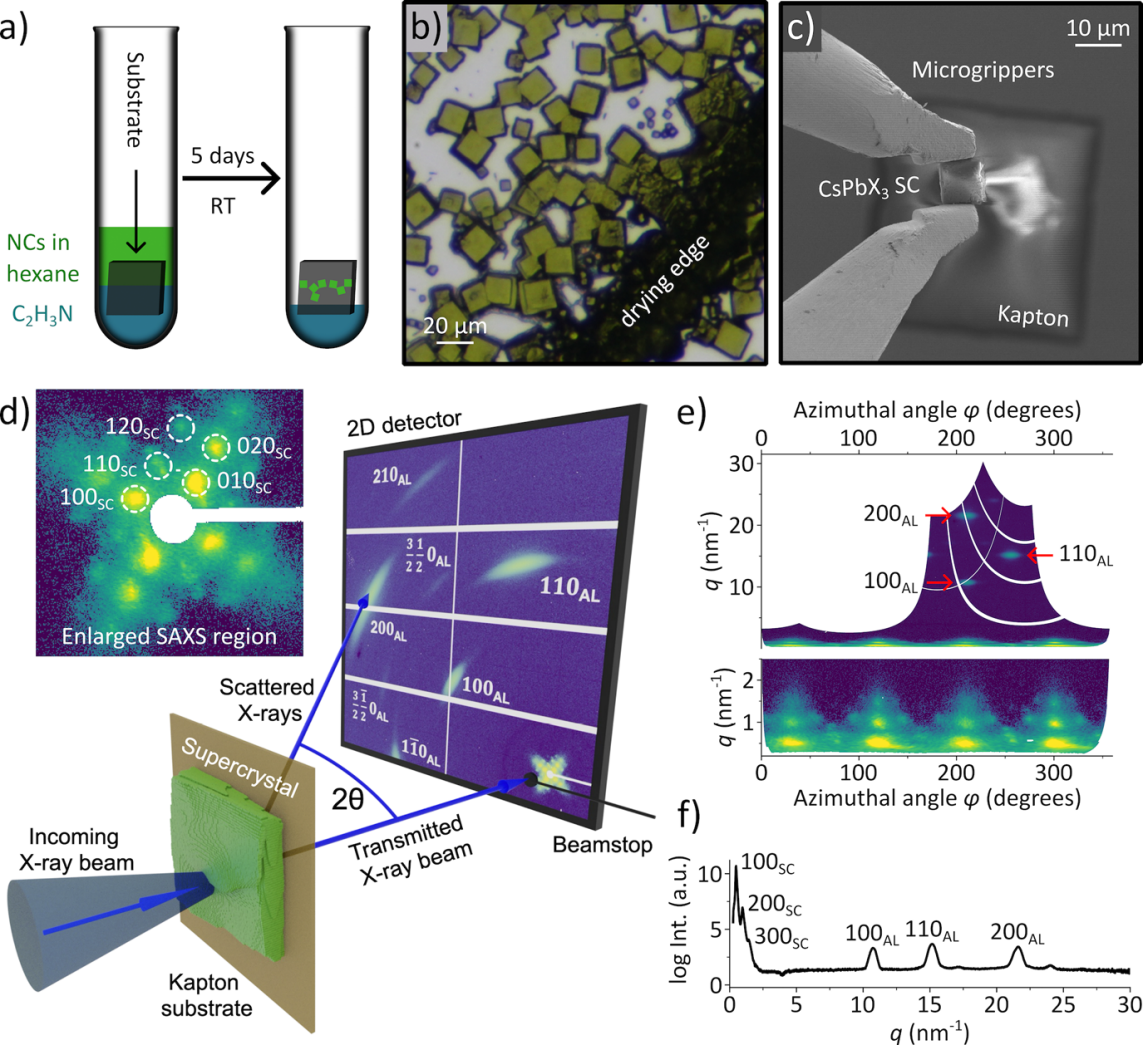
Overview of the sample preparation and spatially resolved structural characterization of CsPbX_3_ SCs prepared by a two-phase layer diffusion technique. (a) Assembly of SCs from NCs at room temperature (RT) using a two-layer phase diffusion technique. (b) Optical micrograph of the obtained SCs at the drying edge. (c) SEM image of microgrippers being used to transfer a SC from the growth substrate onto a Kapton substrate for X-ray studies. (d) Schematic of the X-ray nanodiffraction experiment in transmission geometry. The inset shows a closeup of the SAXS region of a representative diffraction pattern recorded from a CsPbBr_3_ SC in logarithmic scale. The diffraction peaks from the atomic lattice (AL) and supercrystals (SCs) are labeled. (e) The scattered X-ray intensity *I*(*q*, φ) in polar coordinates of the representative diffraction pattern. (f) Radial profile of the scattered logarithm of intensity of the representative CsPbBr_3_ SC diffraction pattern.

We determine the structure of isolated SCs on Kapton by X-ray nanodiffraction, using the experimental scheme in Figure 1d.^21,40,41^ Briefly, the setup allows simultaneous recording of the first three Bragg peaks of the perovskite atomic lattice (AL, *q* = 10–30 nm^–1^) and the reflections of the superlattice of NCs. Typical wide-angle X-ray scattering (WAXS) is displayed in Figure 1d with indexing according to a pseudo-cubic notation,^21^ which we assume for simplicity. The weak half-integer diffraction peaks in the pseudo-cubic notation appear due to the fact that CsPbBr_3_ actually has an orthorhombic crystal structure, in which the PbBr_6_ octahedra are slightly tilted.^15,42,43^ Figure 1d also displays a closeup of the small-angle X-ray scattering (SAXS) region with indexing according to a simple cubic structure of the SC. In Figure 1e, we present the same data in polar coordinates *I*(*q*, φ), where the origin corresponds to the position of the transmitted beam, and Figure 1f displays a radial profile of the scattering recorded from the CsPbBr_3_ SC. Well-defined diffraction peaks in SAXS indicate a highly ordered structure of the SC, while the presence of the Bragg peaks from the atomic lattice in WAXS suggests that the orientation of individual NCs is aligned with the crystallographic directions of the superlattice.^41,44^ By fitting the diffraction peaks in polar coordinates with 2D Gaussian functions, one can extract the structural parameters of the superlattice and the atomic lattice. Details of the data analysis and fitting procedure are provided in section S3 of the SI. The Bragg peaks of the atomic lattice are observed at *q* = 10.8 nm^–1^ (100_AL_), *q* = 15.2 nm^–1^ (110_AL_), and *q* = 21.6 nm^–1^ (200_AL_) corresponding to an average pseudo-cubic atomic lattice unit cell parameter of 5.84 Å, consistent with earlier reports.^42,45^ Weak fringes around the Bragg peaks arise from the cubic shape of individual NCs.^15^ The presence of these fringes indicates that the NCs within the volume of the SC illuminated by the focused X-ray beam have a narrow size distribution; otherwise, the fringes would be smeared or absent.

Figure 2a shows the spatially averaged SAXS pattern obtained from a CsPbBr_3_ SC. Smeared diffraction peaks indicate that the parameters of the SC structure (Figure 2b) exhibit spatial variations, which were proven by spatially resolved analysis. We scan the CsPbBr_3_ SC sample on a 61 × 61 grid with a step size of 400 nm and obtain the spatially resolved map of the SAXS intensity in Figure 2c. Each pixel of this map corresponds to the integrated intensity of the SAXS signal from a specific diffraction pattern. For the points where the SAXS intensity is high enough, the analysis of the SAXS peaks allows one to extract the local values of the structural parameters schematically described in Figure 2b. The angular positions of the four first order SAXS peaks 100_SC_ and 010_SC_ provide information on the rhombicity^46^ and orientation of the superlattice, which are characterized by the angles γ and ψ, respectively. The spatially resolved map of the angle γ (Figure 2d) shows that there are only small deviations from a cubic lattice, for which γ = 90°. The angle γ exhibits spatial variations within ±10° without a clear geometrical trend, indicating a high degree of orientational order within the SC. Figure 2e shows that the orientation of the superlattice, described by the angle ψ (see the definition of ψ in Figure 2b), is also well-preserved within the whole SC since ψ exhibits only small variations within ±5° when scanned across the SC.

**Figure 2:**
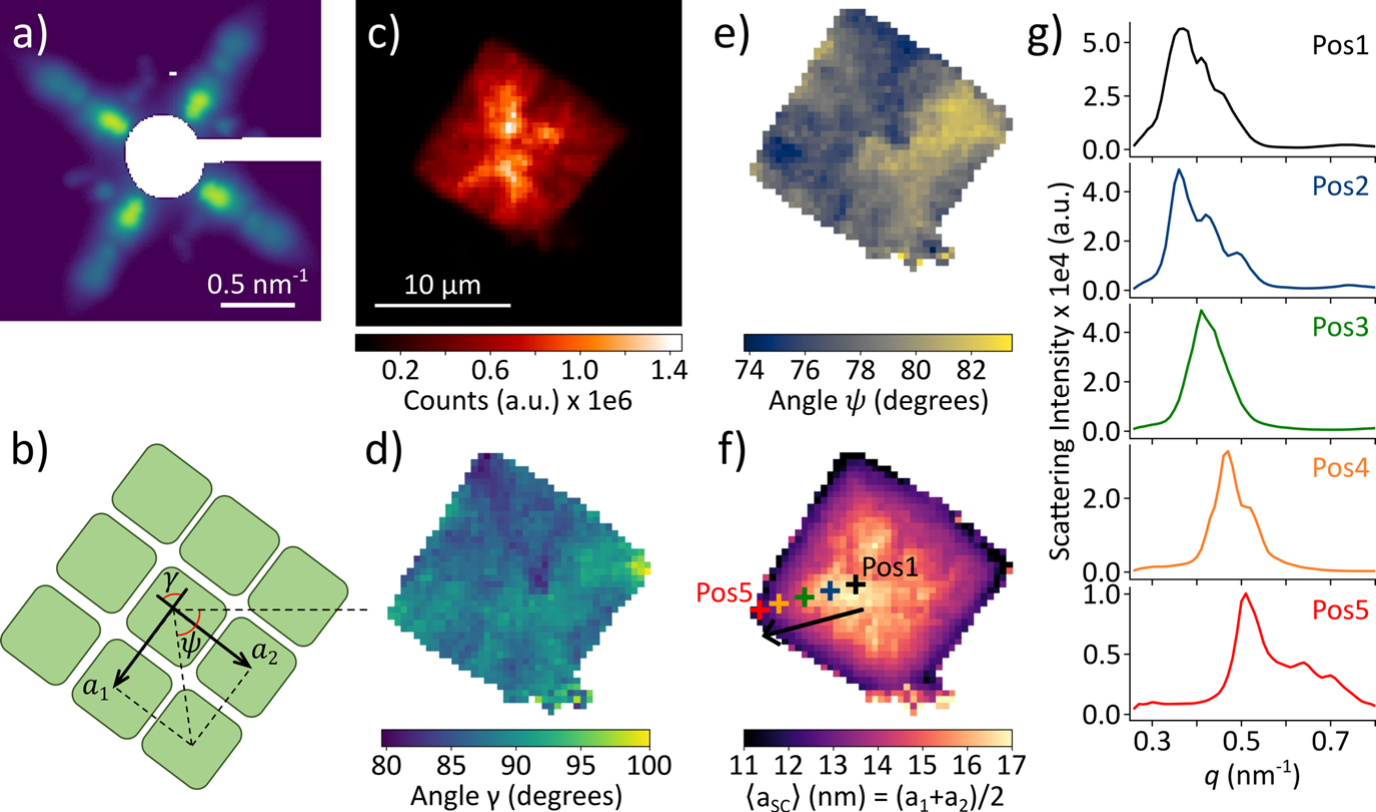
Spatially resolved structure of a CsPbBr_3_ SC revealed by X-ray nanodiffraction. (a) Spatially averaged SAXS pattern of the SC (logarithmic scale). (b) Definition of the structural parameters of the SC: the unit cell parameters *a*_1_, *a*_2_, and γ of the SC and its orientation ψ with respect to a horizontal reference axis. (c) Intensity map of the sample obtained by integrating the intensity of the SAXS region for *q* < 2 nm^–1^. (d) Spatial map of the angle γ characterizing the rhombicity of the SC structure. (e) Spatial map of the orientation of the SC lattice ψ. (f) Spatial map of the average SC unit cell parameter with five distinct positions on a line from the SC center to its edge. (g) Radial profiles of the first order SAXS peaks corresponding to the five positions specified in (f).

The radial position of the SAXS peaks allows one to extract the two lattice parameters *a*_1_ and *a*_2_ and calculate ⟨*a*_SC_⟩ = (*a*_1_ + *a*_2_)/2, representing the average center-to-center distance between neighboring NCs within the SC. The parameter ⟨*a*_SC_⟩ encompasses the NC size and the spacing between them, and it can be interpreted as the lattice parameter of the SC. The spatially resolved map of ⟨*a*_SC_⟩ is displayed in Figure 2f. We observe a continuous increase of ⟨*a*_SC_⟩ by approximately 6 nm from the edges of the SC toward the center. Figure 2f displays five distinct positions on a line spanning from the center of the SC (Pos1) to the edge (Pos5). The radial profiles of the first order SAXS peaks (Figure 2g) at these positions reveal a continuous shift toward higher *q*-values, corresponding to smaller ⟨*a*_SC_⟩, when approaching the edge of the crystal. The SAXS measurement in the center of the SC with the first peak at *q* = 0.37 nm^–1^ corresponds to an NC center-to-center distance of 16.98 nm, while at the edge the NC center-to-center distance reduces to 12.32 nm (the first SAXS peak at *q* = 0.51 nm^–1^). The presence of larger NCs in the center of the SC was not expected since the stock solution only contained smaller NCs with a relatively narrow size dispersion, namely, 7.45 ± 0.89 nm. (The characterization of the NCs in all stock solutions used in this work is summarized in Table S1 in the SI.) Moreover, the selectivity of the self-assembling SC against incorporating too large or too small NCs typically results in an even narrower size distribution of the NCs in the SC than in the original stock solution.^1,47^ The unusual gradient of NC sizes within a single SC will be investigated in more detail further below.

The same trends, particularly the presence of large NCs in the center of a SC, were observed on other SCs formed by NCs of similar size but different perovskite materials. These results are shown in Figure 3 for CsPbBr_2_Cl (Figure 3a–d) and CsPbCl_3_ (Figure 3e–h). We note that the CsPbCl_3_ SCs were grown directly on Kapton, while the CsPbBr_2_Cl SCs were relocated from a Si substrate in the same manner as the CsPbBr_3_ SC shown in Figure 2.

**Figure 3:**
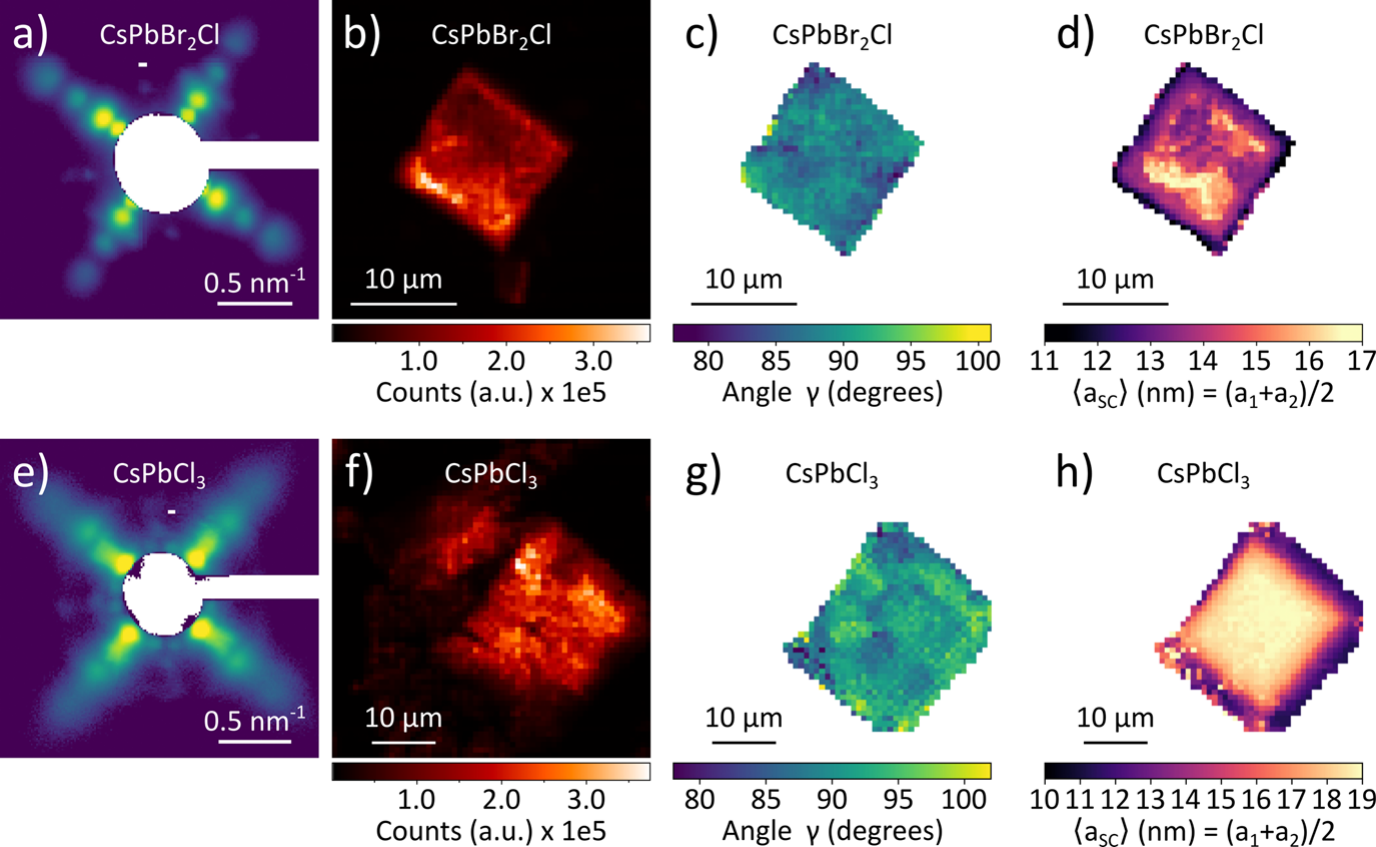
Spatially resolved structures of CsPbBr_2_Cl and CsPbCl_3_ supercrystals. (a, e) Intensity maps of the samples obtained by integrating the intensity of the SAXS region for *q* < 2 nm^–1^. (b, f) Spatially averaged SAXS patterns of the SCs (logarithmic scale). (c, g) Spatial maps of the angle γ between the SC lattice parameters *a*_1_ and *a*_2_. (d, h) Spatial maps of the SC unit cell parameters, calculated from the radial positions of the first order SAXS peaks.

Two representative SCs of CsPbBr_2_Cl and CsPbCl_3_ were raster-scanned with a focused X-ray beam on a 61 × 61 grid with a step size of 500 nm for CsPbBr_2_Cl and 833 nm for CsPbCl_3_. The average atomic lattice constants of 5.78 and 5.61 Å, determined from the position of the WAXS peaks, are consistent with the expected smaller atomic unit cells for CsPbBr_2_Cl and CsPbCl_3_, respectively.^48,49^

The SAXS patterns averaged over the full crystals (Figure 3a,e) are comparable to the one for the CsPbBr_3_ NC in Figure 2a. The X-ray microscopy images in Figure 3b,f, obtained by using the integrated SAXS signal, display similar sizes and shapes of the SCs as those for CsPbBr_3_ (Figure 2c). Similarly, the maps of angle γ shown in Figure 3c,g reveal only slight deviations of the superlattice from an ideal cubic structure. Most importantly, the spatial maps of ⟨*a*_SC_⟩ in Figure 3d,h reveal the same increasing trend toward the center of the SC with a total difference of 6 nm for CsPbBr_2_Cl and 9 nm for CsPbCl_3_.

We argue that the apparent increase in NC size toward the center found in the X-ray scattering experiment should also be visible by electron microscopy. We choose scanning electron microscopy (SEM) for this purpose, as the large SC thickness prohibits microscopy in transmission geometry. We refrain from analyzing the top layer of a SC since this layer is prone to aging with potentially unrepresentative results (see Figure S9 in the SI). Instead, we noticed that frequently the removal of a SC from the crystallization substrate leaves the bottom layer of NCs behind due to its strong adhesion to the substrate. This bottom layer constitutes an ideal specimen for SEM analysis, as it can be freshly measured with minimal aging. Figure 4 shows the results obtained on a CsPbBr_2_Cl SC, and the analogous studies of a CsPbBr_3_ SC are given in section S2 of the SI. We selected three regions of interest (Figure 4a,b): one close to the former edge of the SC (blue), an intermediate region (green), and a region close to the former center (yellow). The corresponding high-resolution SEM images and fast Fourier transforms (FFTs) of these regions are displayed in Figure 4c. From the positions of the first maxima in the FFTs at Δ*k* = 0.083, 0.068, and 0.053 nm^–1^, we deduce the values of ⟨*a*_SC_⟩ = 1/Δ*k*, increasing from 12.0 nm at the edge to 18.9 nm in the center. These findings corroborate our X-ray nanodiffraction results.

**Figure 4:**
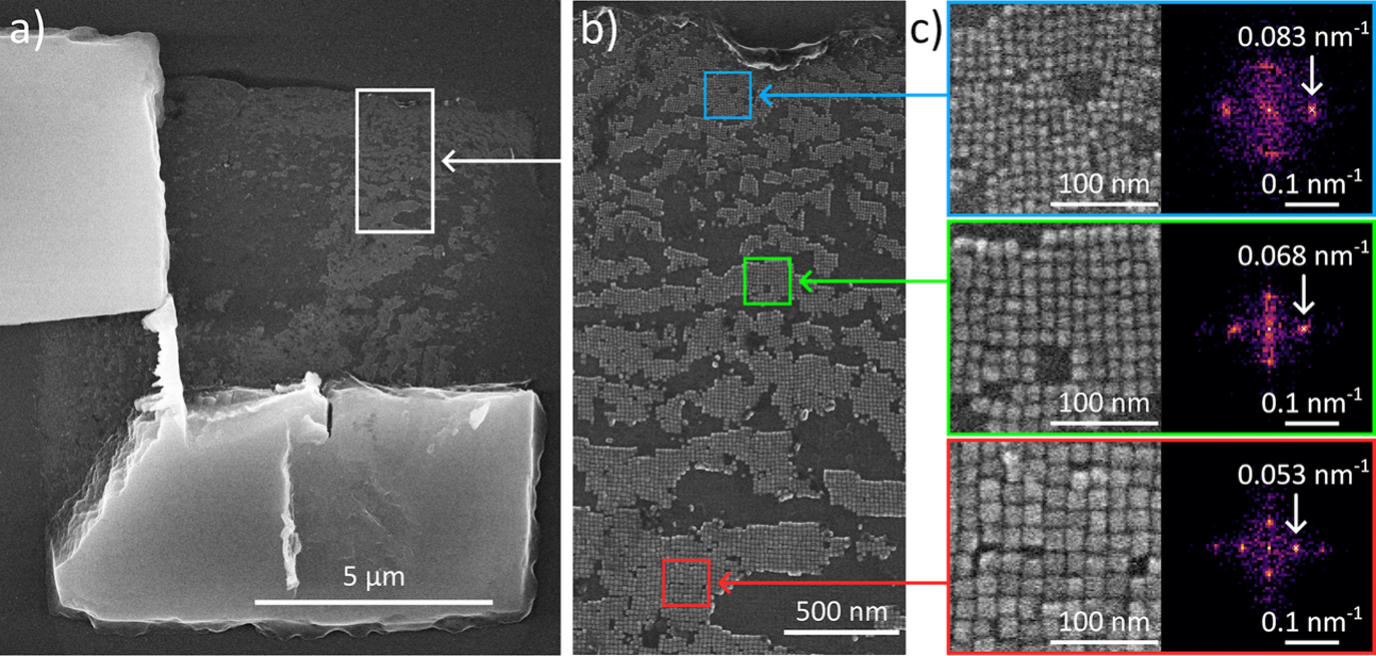
SEM images of a CsPbBr_2_Cl SC, partially removed from the substrate. (a) SEM image of a CsPbBr_2_Cl SC, assembled via the two-layer phase diffusion technique and broken in half using microgrippers. Residual nanocrystals form an incomplete monolayer, which remains adhered to the substrate. (b) Closeup of the residual nanocrystal monolayer in the area marked by the white rectangle in (a). Three regions of interest are highlighted: NCs near the edge (blue), NCs in an intermediate region (green), and NCs near the SC center (red). (c) Magnified views of the nanocrystals from the highlighted regions in (b) and their respective Fourier transformations. The distances in reciprocal space from the zero-frequency point are labeled in the Fourier plots.

To check the NC size distribution along the vertical direction, we conduct another SEM experiment, where we intentionally cleave a SC and scan the variance in NC size at the freshly cleaved SC facet with an electron beam along the height of the crystal (see section S2.2 of the SI). We observe no significant trend in the NC size along the vertical direction (Figures S14 and S15 in the SI), while the change in lateral direction is apparent on this sample (Figure S16 in the SI). This implies that the gradual increase in ⟨*a*_SC_⟩ toward the center is mostly limited to the in-plane direction, indicating potentially anisotropic growth of the SCs.

We hypothesize that the unexpected growth and size gradient found for all NC compositions are the result of the two-layer phase diffusion technique, which is typically not applied for the crystallization of perovskite SCs. To test this, we prepare SCs from CsPbBr_3_ NCs using the commonly applied “slow evaporation” technique without the use of an antisolvent. These NCs have a smaller size, namely, 6.4 ± 0.6 nm (see Materials and Methods and section S1.4 of the SI). As illustrated in Figure 5a, SCs obtained by the slow evaporation technique exhibit a wide range of lateral sizes, from 2 × 2 μm^2^ to 30 × 30 μm^2^ with sharp polygonal outlines and thicknesses between 0.5 and 1 μm. All attempts to relocate SCs in this manner with microgrippers failed, as they deformed immediately upon contact (Figure 5b).

**Figure 5:**
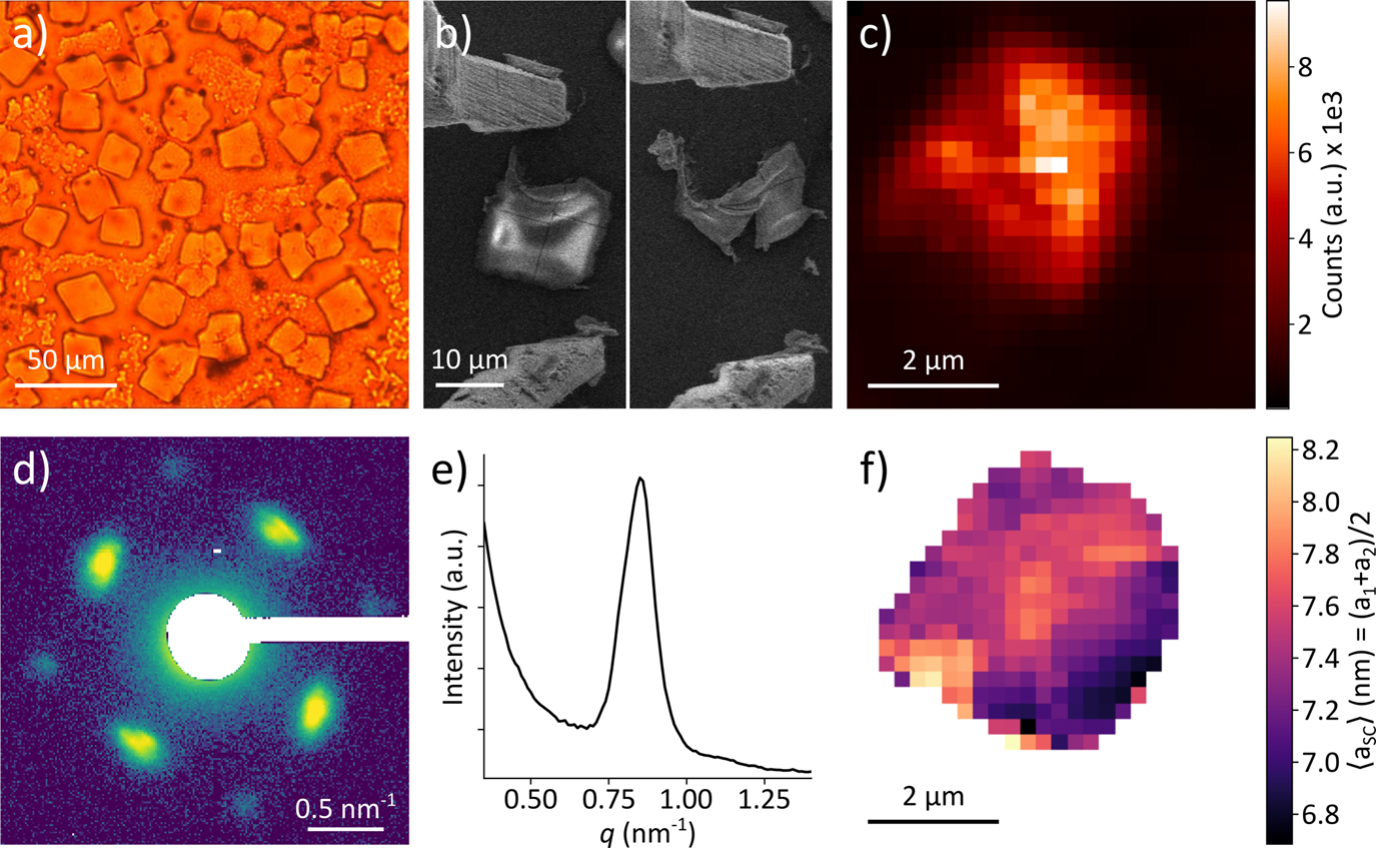
Sample preparation and spatially resolved structural characterization of a CsPbBr_3_ SC assembled by slow solvent evaporation. (a) Optical micrograph of the SCs on a Kapton substrate. (b) SEM images showing the deformation of a SC, self-assembled via slow evaporation, caused by an attempt at mechanical manipulation using microgrippers. (c) Intensity map of the sample obtained by integrating the intensity of the SAXS region for *q* < 2 nm^–1^. (d) Spatially averaged SAXS pattern of the SC (logarithmic scale). (e) Radial profile of the first order SAXS signals of the average SAXS pattern. (f) Spatial map of the SC unit cell parameter, calculated from the radial positions of the first order SAXS peaks.

To obtain insight into the structure, we performed a spatially resolved X-ray study of a representative SC prepared by slow evaporation on Kapton substrates^50^ (Figure 5c). The diffraction patterns were collected over a 26 × 26 grid with 240 nm steps. The SAXS pattern averaged over the entire SC exhibits discrete SAXS peaks, indicating structural homogeneity (Figure 5d). In agreement with this, the average radial SAXS profile shows a single narrow peak at *q* = 0.85 nm^–1^, corresponding to ⟨*a*_SC_⟩ = 7.39 nm (Figure 5e). The spatially resolved map of ⟨*a*_SC_⟩ in Figure 5f, calculated from the radial positions of the first order SAXS peaks, reveals that there is little spatial variation from this average and no overall trend from the edge to the center. A comparison with the NC size in solution (6.4 ± 0.6 nm) and ⟨*a*_SC_⟩ = 8.4 nm obtained from electron microscopy (Figure S8 in the SI) suggests that no significant NC growth takes place during assembly with the slow evaporation technique. These results are in stark contrast to those obtained for SCs assembled using the two-layer phase diffusion technique, supporting our hypothesis that the presence of the antisolvent acetonitrile is responsible for the NC growth during crystallization. This means that the growth of the NCs in the center of a SC most probably occurs during the self-assembly, and it is not related to the initial polydispersity of the NCs in stock solution.

We argue that due to its polarity, acetonitrile can partially solubilize ligands from the NC surface, leading to the formation of exposed and reactive interfaces. In such an environment, CsPbBr_3_ could potentially be very slightly soluble in acetonitrile, enabling mobility of species and promoting slow recrystallization of NCs. We propose that large NCs grow at the expense of smaller ones as the material redistributes to minimize the surface energy, akin to Ostwald ripening. The observed size gradient suggests that larger NCs are more likely to initiate nucleation and participate in the early stages of SC growth. Introducing a poor solvent raises the chemical potential per NC, making size-dependent interparticle attraction the dominant thermodynamic driving force for crystallization.^12^ The same rationale is applied during size-selective precipitation, where the first fraction typically contains the largest particle diameters.^51^ The presence of clear SAXS peaks indicates that the NCs maintain an ordered assembly within the SC and do not aggregate or stick together uncontrollably. This suggests that the ligand coverage, although potentially reduced, remains sufficient to prevent uncontrolled aggregation of the NCs.

We conclude the analysis by quantifying the mechanical strength of SCs prepared by the two different techniques, which was already apparent from attempts to relocate the SC by microgrippers (Figures 1c and 5b). To this end, we employ nanomechanical mapping using an atomic force microscope (AFM). The main results are displayed in Figure 6, with details of the experiment provided in section S4 of the SI. For the SCs assembled using the two-layer phase diffusion technique, we measured the spatially inhomogeneous Young’s modulus *E* across the SCs (Figure 6a). The Young’s modulus increases toward the center of the SC (Figure 6b). This increase does not correlate with the thickness of the SC, which remains fairly constant. We speculate that the values of the Young’s modulus correlate with the increasing size of the NCs and ⟨*a*_SC_⟩ from the edges toward the center of the SC, which may indicate that there are less soft ligands in the central regions of the SC. In contrast, we observe almost a constant value of the Young’s modulus in the nanomechanical maps of SCs assembled using solvent evaporation (Figure 6c). These SCs have a similar flat profile of the height (Figure 6d) as that for the SCs shown in Figure 6b, but the Young’s modulus does not change dramatically at different positions in the SC, which correlates with the uniform distribution of NC size and ⟨*a*_SC_⟩ (Figure 5f). Most importantly, we observe a clear difference in the absolute values, which is evident for the exemplary SCs shown in Figure 6 as well as other measured SCs (see section S4 of the SI). The mean value and variance for Young’s modulus are *E* = 3.2 ± 1.3 GPa for the SCs fabricated via the two-phase layer diffusion method and *E* = 0.14 ± 0.07 GPa for the SCs fabricated via slow evaporation of the solvent (see details in section S4.5 of the SI). One possible explanation of the approximately 10-fold increase in the hardness of the SC exposed to acetonitrile could be a decreased density of the soft ligand shell surrounding the NCs. However, even for the mechanically robust SCs, the values of Young’s modulus are still almost five times smaller than *E* = 16 GPa in bulk CsPbBr_3_.^52–54^ This indicates that some organic ligands still remain between the individual NCs and prevent them from merging into a single crystal, which is also supported by the X-ray diffraction and SEM measurements.

**Figure 6:**
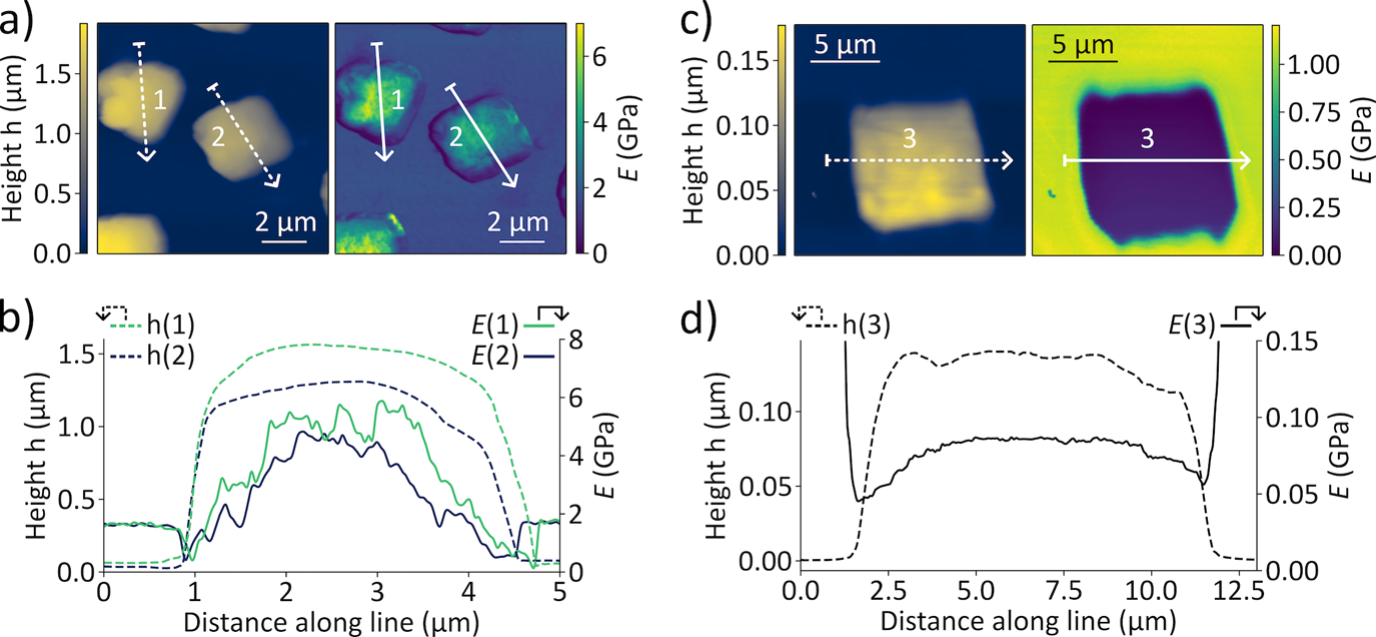
Results of AFM nanomechanical mapping performed on CsPbBr_3_ SCs. (a) 2D maps of the height *h* (left) and Young’s modulus *E* (right) of two SCs produced by the two-layer phase diffusion technique. (b) The height and Young’s modulus along the two lines shown in (a). (c) 2D maps of the height *h* (left) and Young’s modulus *E* (right) of a representative SC fabricated by slow evaporation of the solvent. (d) The height and Young’s modulus along the line shown in (c).

## Conclusions

We attribute the softness of perovskite SCs grown via slow solvent evaporation to the organic ligands surrounding the NCs. These SCs form randomly on substrates and are too soft to manipulate with microgrippers. To overcome this, we present a two-phase layer diffusion method that promotes the self-assembly of NCs into highly ordered and mechanically robust SCs.

We propose that exposure to acetonitrile antisolvent during self-assembly triggers NC growth through dissolution and reprecipitation. Structural analysis using nanofocused X-ray scattering and SEM supports this, revealing a marked increase in NC size toward the SC center, unrelated to the initial size distribution. In contrast, SCs from solvent evaporation show no such gradient. This size variation within a single SC enables systematic studies of size-dependent properties such as light emission and absorption.

Mechanically, SCs from the diffusion method exhibit a Young’s modulus an order of magnitude higher than those from solvent evaporation, making them suitable for manipulation and precise placement. This advancement allows for integrating perovskite SCs into complex microdevices, where positioning near features like electrodes or waveguides is crucial for functionality and further research.

## Materials and Methods

### Synthesis of CsPbX_3_ NCs and Assembly of NCs into SCs

#### Chemicals

1-Octadecene (ODE), technical grade, 90%, Sigma-Aldrich; oleic acid (OA), 97%, Acros Organics; oleylamine (OLA), 80–90%, Acros Organics; cesium carbonate (Cs_2_CO_3_), 99.99% (trace metal basis), Acros Organics; lead­(II) chloride (PbCl_2_), 99.999% (trace metal basis), Sigma-Aldrich; lead­(II) bromide (PbBr_2_), 99%, Acros Organics; lead­(II) acetate trihydrate (PbOAc), 99.99% (trace metal basis), Sigma-Aldrich; zinc­(II) bromide (ZnBr_2_), ≥99.99%; *n*-hexane, 97% extra dry over molecular sieve, AcroSeal, Acros; and acetonitrile, 99.9% extra dry over molecular sieve, AcroSeal, Acros. All chemicals were used as purchased.

#### CsPbX_3_ NCs

CsPbX_3_ NCs with a size of approximately 8 nm were synthesized by a hot-injection method according to a slightly modified literature method by Dutta et al.^55^ First, 97 mg (0.3 mmol) of Cs_2_CO_3_ and 227 mg (0.6 mmol) of PbOAc were dissolved in a mixture of 30 mL of 1-octadecene and 3 mL of oleic acid in a 50 mL three neck flask and degassed under vacuum at 120 °C for 2 h. Subsequently, the temperature was increased to 180 °C for CsPbCl_3_ NCs under nitrogen atmosphere and to 240 °C for CsPbBr_2_Cl and CsPbBr_3_ NCs, respectively. Depending on the halide composition of the NCs, 3 mL of an OLA-HL precursor solution (1.1 M) was swiftly injected. After the synthesis, the NCs were separated by centrifugation at 10 000 rpm for 10 min. The supernatant was discarded, and the crude precipitate was centrifuged again at 10 000 rpm for 10 min. Resulting supernatant traces were removed via syringe, and the precipitate was dissolved in 3 mL of hexane. The dissolved particles were filtered through a 0.2 μm PTFE syringe before they were stored in a glovebox.

#### Smaller CsPbBr_3_ NCs

The synthesis and isolation of the smaller CsPbBr_3_ NCs (with a size of approximately 6 nm) were performed following the procedure described by Dong et al.^56^ Briefly, 0.4 mL of cesium oleate stock solution in 1-octadecene (0.16 M) was injected into a mixture of PbBr_2_ (75 mg, 0.2 mmol) and ZnBr_2_ (120 mg, 0.8 mmol) solubilized in the presence of oleic acid (2 mL) and oleylamine (1 mL) in 1-octadecene at 165 °C. After the synthesis, the NCs were isolated in several steps using centrifugation with anhydrous ethyl acetate as an antisolvent. The resulting NCs were redispersed in anhydrous toluene to yield a stock solution for self-assembly.

#### SC Growth via Two-Layer Diffusion Technique

The SCs were grown on silicon wafers (10 × 10 mm^2^) under a nitrogen atmosphere. The substrate to be coated was placed in a test tube (10 mm in diameter), located in a 50 mL centrifuge tube. Afterward, 600 μL of an approximately 2 μM solution of the NCs in hexane was under layered with 600 μL of acetonitrile. The centrifuge tube was sealed, covered with aluminum foil, and allowed to rest for 5 days at room temperature. At the end of the crystallization time, the remaining solvent residues were removed, and the substrates were dried for 2 h. The dried samples were inspected under an optical microscope for a visual confirmation of the formation of microscopic SCs.

#### SC Growth via Evaporation Technique

The SCs were grown on square Kapton pieces (1.5 × 1.5 cm^2^, thickness of 0.125 mm, Kapton 500HN, CMC 70125 by CMC Klebetechnik) or silicon wafers (10 × 10 mm^2^). The NC stock solution was diluted with anhydrous toluene in a 1:3 proportion, and 40 μL of it was placed on top of the substrate lying flat inside a Petri dish. The covered Petri dish was left to dry for several hours under an air or nitrogen atmosphere until all solvent evaporated. The dried samples were inspected under an optical microscope for visual confirmation of the formation of microscopic SCs.

### Scanning Electron Microscopy and Mechanical Manipulation of SCs

The crystals were transferred using MM3A-EM SEM-compatible micromanipulators (Kleindiek Nanotechnik) equipped with MGS2-EM microgrippers (Kleindiek Nanotechnik). The transfer process was conducted using an LEO Gemini 1550 VP scanning electron microscope (Zeiss). High-resolution scanning electron micrographs were recorded using a SU8030 (HITACHI) with a resolution of approximately 1.2 nm.

### Atomic Force Microscopy and Nanomechanical Mapping

Height and mechanical mapping measurements were performed by using a MultiMode 8 HR atomic force microscope (Bruker). RTESPA-75 probes (Bruker) were used for data acquisition. Prior to measurements, the instrument was calibrated for the deflection sensitivity, tip radius, and spring constant. The deflection sensitivity was calibrated by using the SAPPHIRE-12 M calibration standard (Bruker). The tip radius was measured using a Leo Gemini 1550 VP scanning electron microscope (Zeiss). The spring constant was calculated using the Sader method.^57^ Samples were prepared on 0.02 mm-thick Muscovite mica substrates (KaI_3_Si_3_O_10_(OH)_2_, Micro to Nano). The Nanoscope Analysis 3.0 software (Bruker) and the open-source software Gwyddion were used in the analysis of the data.^58^

### Optical Measurements

Absorbance spectra were acquired by using a UV–vis–NIR spectrometer (Cary 5000, Agilent Technologies). Emission spectra were acquired on a fluorescence spectrometer (PerkinElmer FL8500).

### X-ray Nanodiffraction Experiments

Nanodiffraction experiments were performed at the Coherence Applications beamline P10 of the PETRA III synchrotron source at DESY using the GINIX nanofocusing end station.^59,60^ The X-ray beam was focused to approximately a 240 nm (vert.) × 320 nm (hor.) size (fwhm). To minimize the X-ray fluorescence background, the photon energy was chosen to be *E* = 13 keV (λ = 0.0954 nm), which is just below the L-III absorption edge of lead. The diffraction patterns were recorded by an EIGER X 4M detector placed 398 mm behind the sample in transmission geometry. The specific scan parameters for the spatially resolved maps are provided alongside the measurements in the figure captions of the main text. At each spatial position the acquisition time was 1 s, which ensured a high signal-to-noise ratio while preventing radiation damage of the sample. The scattering background from a pure Kapton film was subtracted from every collected pattern.

## Supplementary Material



## Notes

Nanocrystal characterization in solution and supercrystals, details of the sample preparation, analysis of the X-ray nanodiffraction data, and details of the AFM measurements (PDF)

## Abbreviations

NC: nanocrystal
SC: supercrystal
AL: atomic lattice
AFM: atomic force microscopy
SEM: scanning electron microscopy
FFT: fast Fourier transform
fwhm: full width at half-maximum
