# Supplementary material for: Mechanically Robust Supercrystals from Antisolvent-Induced Assembly of Perovskite Nanocrystals
Source: ACS Nano. 2025 Jul 9;19(28):26117–26. doi: 10.1021/acsnano.5c07289 (PMC12291593; doi:10.1021/acsnano.5c07289)
Supplement: Supplementary file 1 [file nn5c07289_si_001.pdf]

Supporting Information

Mechanically robust supercrystals from antisolvent-induced assembly of perovskite nanocrystals

Jonas L. Hiller,[†,||] Robert Thalwitzer,[†,||] Ata Bozkurt,[†] Matheus Gomes Ferreira,[‡] Richard Hodak,[†] Fabian Strauß,[†] Elke Nadler,[†] Gerard N. Hinsley,[¶] Bihan Wang,[¶] Kuan Hoon Ngoi,[¶] Witold Rudzinski,[§] Ekaterina Kneschaurek,[#] Wojciech Roseker,[¶] Michael Sprung,[¶] Dmitry Lapkin,[#] Dmitry Baranov,[‡] Frank Schreiber,[#] Ivan A. Vartanyants,[¶] Marcus Scheele,[,†] and Ivan A. Zaluzhnyy[*,#]*

† Institute for Physical and Theoretical Chemistry, University of Tübingen, Auf der Morgenstelle 18, 72076 Tübingen, Germany

‡ Division of Chemical Physics and NanoLund, Department of Chemistry, Lund University, P.O. Box 124, SE-221 00 Lund, Sweden

¶ Deutsches Elektronen-Synchrotron DESY, Notkestraße 85, 22607, Hamburg, Germany

§ AGH University of Krakow, al Adama Mickiewicza 30, 30-059 Kraków

# Institute of Applied Physics, University of Tübingen, Auf der Morgenstelle 10, 72076, Tübingen, Germany

|| These authors contributed equally to this work

* marcus.scheele@uni-tuebingen.de

* ivan.zaluzhnyy@uni-tuebingen.de

Table of Contents

SECTION S1: PROPERTIES OF THE CSPBX$_3$ NANOCRYSTALS IN SOLUTION 3

S1.1 – CsPbBr$_3$ NCs 3
S1.2 – CsPbBr$_2$Cl NCs 5
S1.3 – CsPbCl$_3$ NCs 6
S1.4 – Smaller CsPbBr$_3$ NCs 8

SECTION S2: PROPERTIES OF THE CSPBX$_3$ NANOCRYSTALS IN THE SUPERCRYSTALS....... 10

S2.1 – Analysis of NC size in SCs, interparticle distance in SCs, and the dimensions of SCs. 10
S2.2 – Cross-section along the vertical and horizontal axis of a SC 13

SECTION S3: X-RAY NANODIFFRACTION DATA ANALYSIS 16

S3.1 – Processing raw data 16
S3.2 – Transforming patterns to polar coordinates 17
S3.3 – Generating spatially-resolved maps of the sample 19
S3.4 – Obtaining the average diffraction pattern of the sample 20
S3.5 – Selecting individual peaks for fitting from the average diffraction pattern 21
S3.6 – Fitting the SAXS and WAXS Bragg peaks 23
S3.7 – Calculating structural properties of NCs and SCs from the diffraction peaks 24

SECTION S4: ATOMIC FORCE MICROSCOPY 25

S4.1 – Determination of the Young modulus 25
S4.2 – Calibration of the deflection sensitivity 25
S4.3 – Calibration of the spring constant 26
S4.4 – Measurement of the tip radius 28
S4.5 – Statistical representation of the Young moduli of different SCs 28

REFERENCES....... 30

Section S1: Properties of the $CsPbX_3$ nanocrystals in solution

Based on the findings presented in the main manuscript of an increasing supercrystal (SC) lattice parameter towards the center of the SCs assembled via the two-layer diffusion technique, it is essential to characterize the nanocrystals (NCs) in the stock solutions, before they are used to fabricate the SCs. The samples of the NCs with the compositions $CsPbBr_3$, $CsPbCl_3$, and $CsPbBr_2Cl$, presented in subsections S1.1 to S1.3, are from the exact stock solutions used to prepare the SCs for the synchrotron measurements. In contrast, the small quantum-confined $CsPbBr_3$ NCs in subsection S1.4 originate from a different batch than the one used to prepare the SCs for the nanodiffraction experiment. However, since both batches were synthesized using the same method and parameters, they exhibit nearly identical optical properties and a comparable size distribution.

All SEM samples were prepared by spin coating 100 µL of the NC stock solution onto a silicon wafer (10×10 mm^2) to characterize the size distribution in the stock solution. From the SEM images the size of the NCs was evaluated in two different ways. The first method is to count the edge length of numerous individual NCs and fit the resulting histograms to the measured values using a Gaussian function. The values determined using this fit method represent the mean value of the size of the NCs, as well as the standard deviation. Due to the presence of numerous organic ligands on the particle surface, the edges of the NCs become indistinct and therefore lead to minor uncertainties in the size determination. Hence, we assume that the actual particle size is underestimated with this method. As a second method, fast Fourier transforms (FFTs) were carried out, the results of which are described in more detail at the respective subsections. The FFT calculates the periodicity of the SEM image, which is directly related to the distance from the center of one NC to the center of its nearest neighbors. This means the calculated distance also includes the ligand shell. Because of the different spatial expansion of the ligand shell, the particle distances also vary, which means that the NC sizes tend to be overestimated.

S1.1 – $CsPbBr_3$ NCs

The optical spectra of the $CsPbBr_3$ NCs were measured by diluting 5 µL of the NC stock solution with 3 mL hexane (**Fig. S1**). The concentration of the NC stock solution was calculated from the absorbance spectrum according to Maes et al. with the absorbance values at 335 nm and 400 nm.[1]

Fig. S2a shows a representative SEM image of a spin coated sample of $CsPbBr_3$ NCs. The edge length of over 1300 NCs were measured, and the corresponding histogram was fitted with a Gaussian distribution (grey curve) resulting in a NC size of 7.45 ± 0.89 nm. In **Fig. S2c**, a four-fold symmetry of the FFT power spectrum is related to the periodic arrangement of the NCs within the film of this sample. The slight broadening of the peaks can be attributed to a non-monodisperse size distribution as well as varying distances between NCs, which tends to cause an overestimation of the NC size. The mean NC size derived from the FFT power spectrum is 10.59 nm.

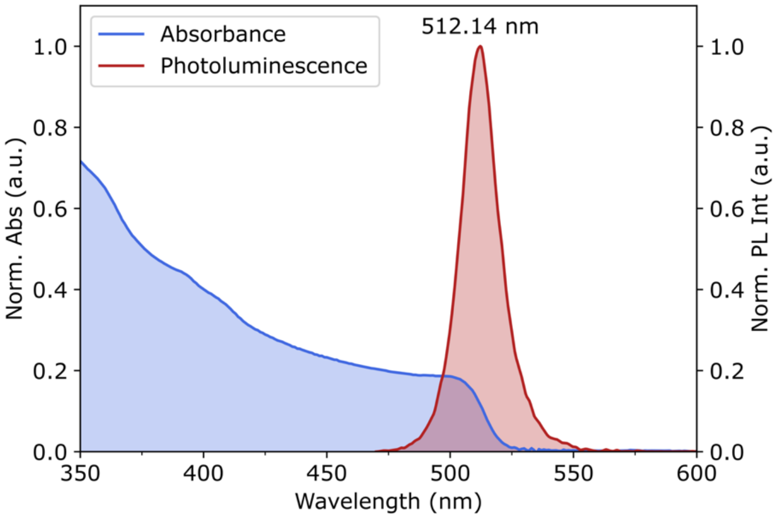

Figure S1: Normalized absorbance and normalized photoluminescence spectrum of $CsPbBr_3$ NCs in hexane. The excitation wavelength λ_{ex} was set to 350 nm. The maximum of the photoluminescence (PL) spectrum can be found at 512.14 nm with a FWHM of the PL peak of 18.87 nm.

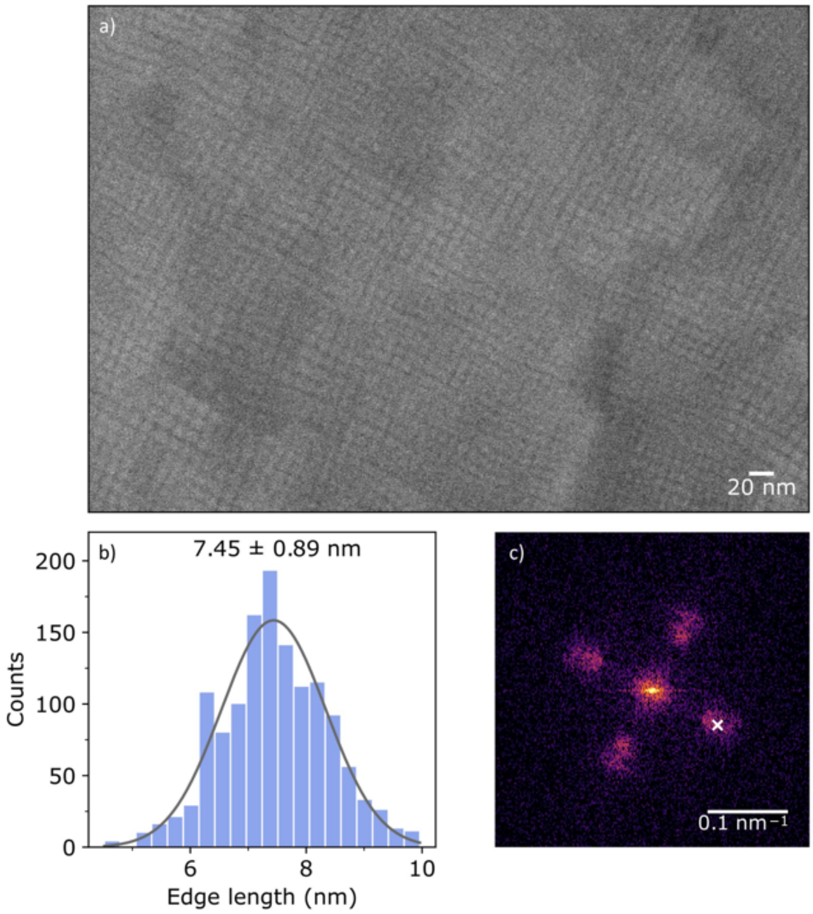

Figure S2: a) SEM image of a thin film of $CsPbBr_3$ NCs deposited on a silicon wafer by spin coating. b) Histogram of the size distribution of the NC resulting from the SEM image. The gray curve represents a Gaussian fit from which the mean size of the NC of 7.45 ± 0.89 nm can be deducted. c) FFT power spectrum of the SEM image. The white cross marks the position corresponding to the average center-to-center distance of 10.59 nm.

S1.2 – $CsPbBr_2Cl$ NCs

The optical spectra of the mixed halide perovskite $CsPbBr_2Cl$ NCs were measured similarly to how it was described in the previous subsection (**Fig. S3**).

The SEM image in **Fig. S4** shows a broader NC size distribution compared to the other samples. Therefore, the edge length of more than 3000 NCs were counted for this sample to better represent the overall homogeneity of the sample, since there are areas with significantly larger particles in addition to areas with very small particles. In total we found 35 NCs ($\sim$1% of all counted particles) with an edge length greater than 15 nm in this overview image. Due to the non-significant number of larger NCs in the sample, we fitted the histogram with a symmetrical Gaussian distribution despite the slightly asymmetrical shape. The larger NCs are included in the fit but are not displayed in the histogram for better visualization. Another challenge that arises from the broader size distribution and the non-periodic arrangement of the NCs in the sample after spin coating is the determination of the FFT power spectrum. An FFT of the entire SEM image in **Fig. S4a** would lead to a circular area that originates from the center of the power spectrum with a boundary at a distance corresponding to the smallest detected NC size. Thus, a more representative FFT power spectrum can only be obtained for smaller selected areas that have a more uniform orientation of the NCs, such as the area marked in blue. For this area, the FFT results in an average NC size of 12.17 nm including the ligand shell.

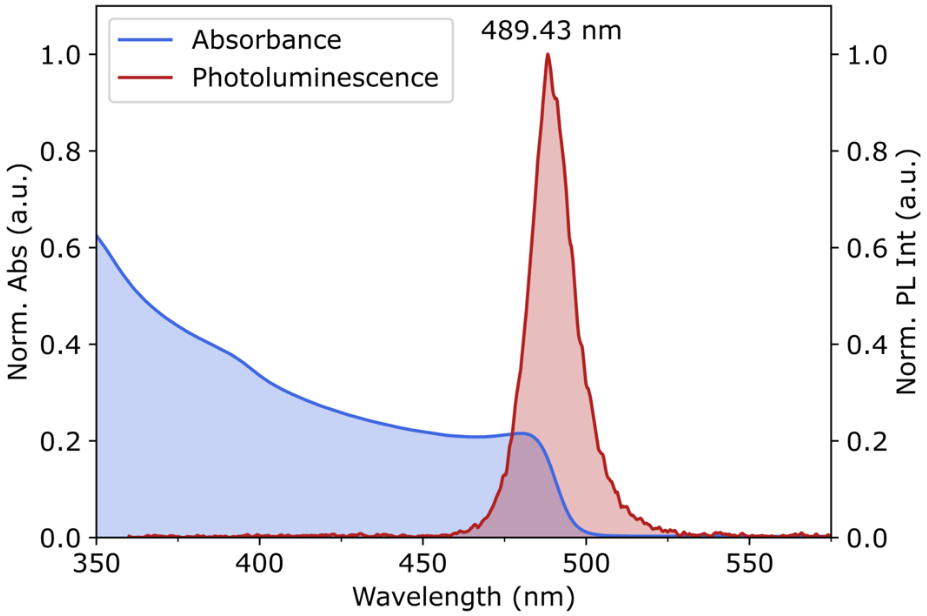

Figure S3: Normalized absorbance and normalized photoluminescence spectrum of $CsPbBr_2Cl$ NCs in hexane (10 µL of stock solution in 3 mL hexane). The excitation wavelength λ_{ex} was set to 350 nm. The maximum of the PL spectrum can be found at 498.43 nm with a FWHM of the PL peak of 16.00 nm.

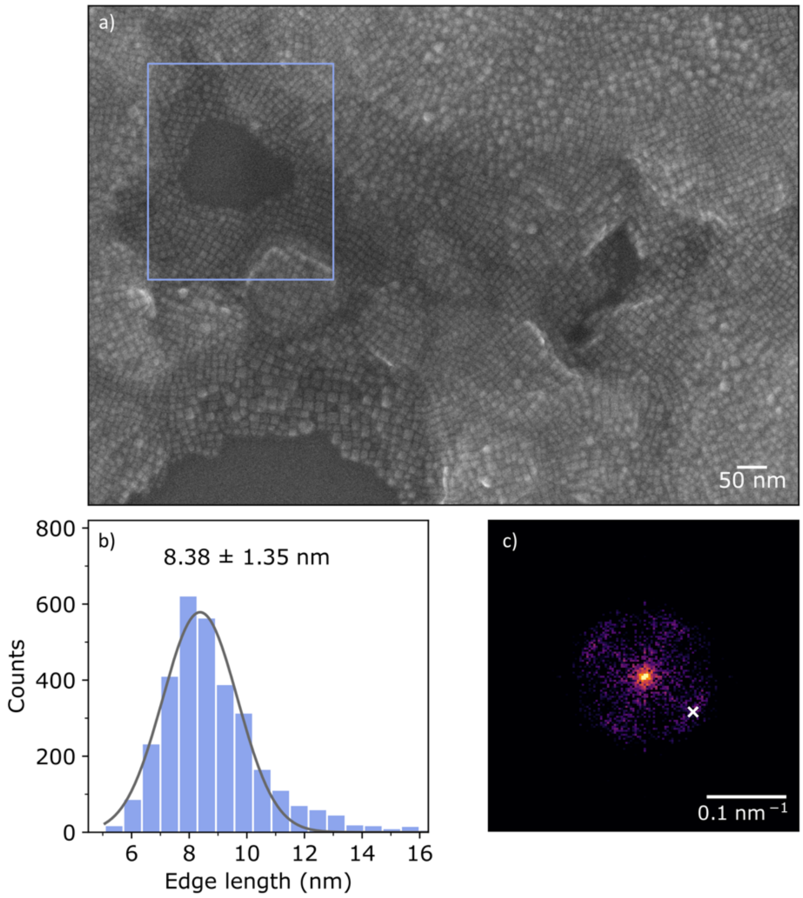

Figure S4: a) SEM image of a thin film of $CsPbBr_2Cl$ NCs deposited on a silicon wafer by spin coating. b) Histogram of the size distribution of the NC resulting from the SEM image. The gray curve represents a Gaussian fit from which the size of the NC of 8.38 ± 1.35 nm can be deducted. c) FFT power spectrum of the selected area in the SEM image with position of average center-to-center distance of 12.17 nm marked with white cross.

S1.3 – $CsPbCl_3$ NCs

Fig. S5 shows the absorbance and photoluminescence spectrum of the blue emitting $CsPbCl_3$ NCs with a PL maximum at 407.05 nm (FWHM: 10.43 nm).

The corresponding SEM image of this sample is displayed in **Fig. S6** together with the resulting size distribution. By measuring the edge length of almost 2000 NCs, the Gaussian fit gives a size distribution of 7.87 ± 1.15 nm whereas the evaluation of the FFT power spectrum leads to an average size of the NCs of 12.43 nm. This deviation between the two methods can be explained by the contribution of ligands to the apparent size of the NCs in SEM, as was already been discussed above. From the SEM image (**Fig. S6a**), a non-symmetric arrangement of the particles can be recognized, as well as areas with different interparticle distances causing broadened peaks in the FFT power spectrum.

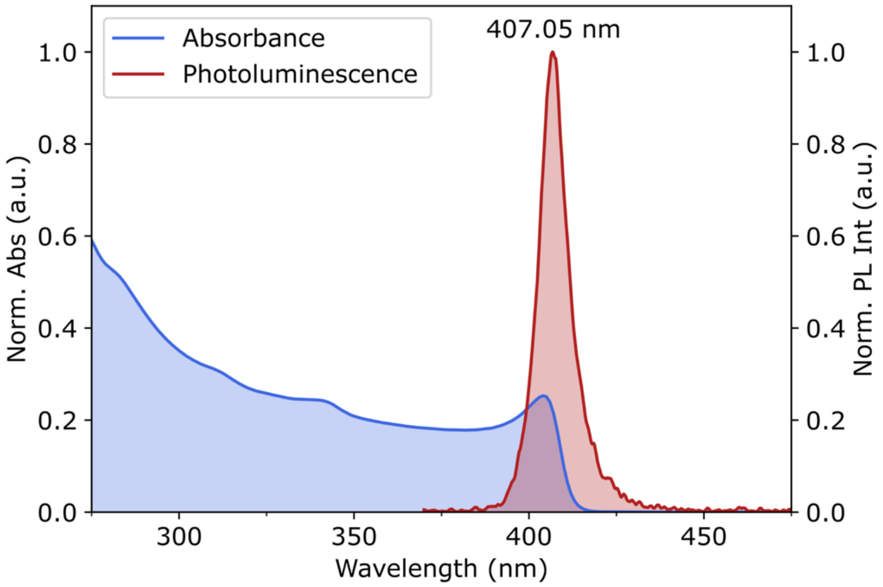

Figure S5: Normalized absorbance and normalized photoluminescence spectrum of $CsPbCl_3$ NCs in hexane (10 µL of stock solution in 3 mL hexane). The excitation wavelength λ_{ex} was set to 350 nm. The maximum of the PL spectrum can be found at 407.05 nm with a FWHM of the PL peak of 10.43 nm.

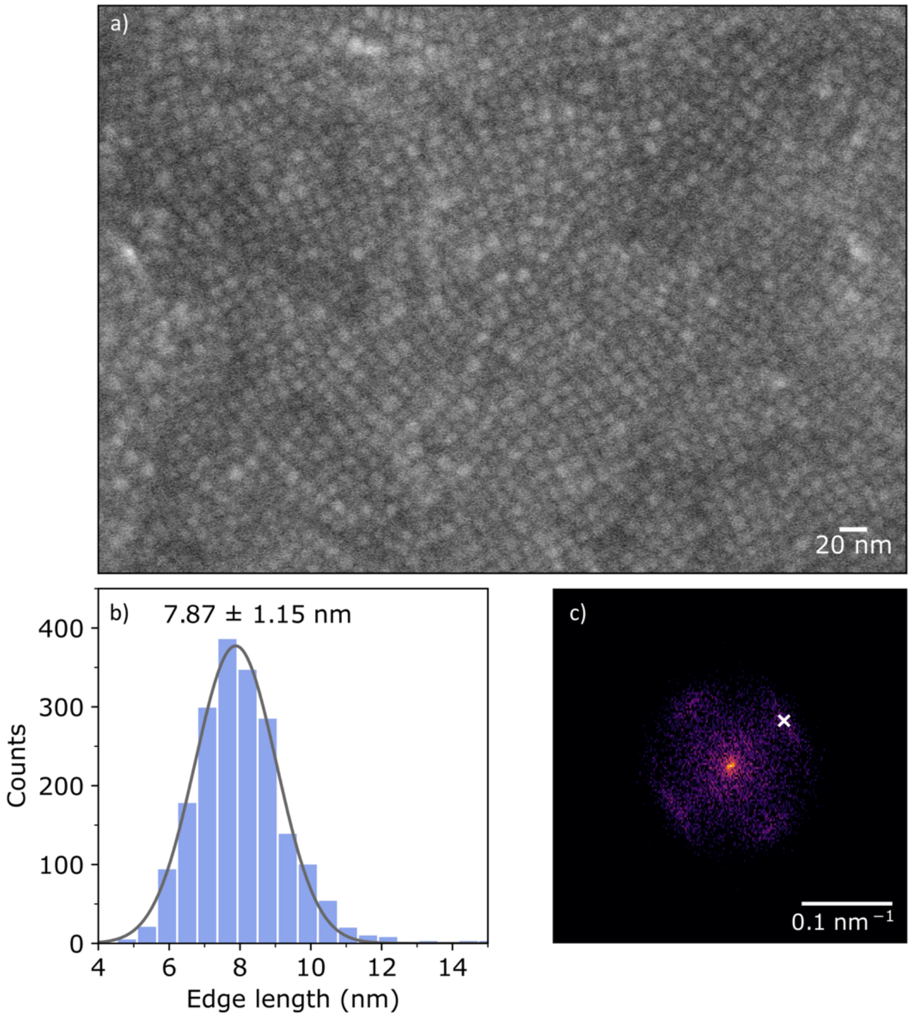

Figure S6: a) SEM image of a thin film of $CsPbCl_3$ NCs deposited on a silicon wafer by spin coating. b) Histogram of the size distribution of the NC resulting from the SEM image. The gray curve represents a Gaussian fit from which the size of the NC of 7.87 ± 1.15 nm can be deducted. c) FFT power spectrum of the SEM image with position of average center-to-center distance of 12.43 nm marked with white cross.

S1.4 – Smaller $CsPbBr_3$ NCs

Additionally, we used a second sample of the exact same stoichiometric composition $CsPbBr_3$, where the size of the NCs is in the quantum confinement regime.[2] These NCs were obtained by a different synthesis route compared to the previous sample (see Materials and Methods section in the main text). As can be seen in the optical spectra in Fig. S7, the appearance of a clear first excitonic transition as well as the position of the PL peak indicate a size of the NCs in the desired quantum confined range. The SEM image in **Fig. S8** and the resulting size distribution of 6.44 ± 0.62 nm by measuring the edge length of the NCs confirms this assumption. From the FFT power spectrum, an average size of 8.44 nm can be derived from the four symmetrically arranged peaks, which points to a narrow size distribution due to the limited expansion. The angular broadening of the FTT peaks originates from the angular disorder in the arrangement of the NCs in the SEM image. Taking the ligand shell into account, the size of the NCs determined from the FFT method agrees very well with the measured size of the edge length.

The sizes of the NCs for all four solutions are summarized in Table S1.

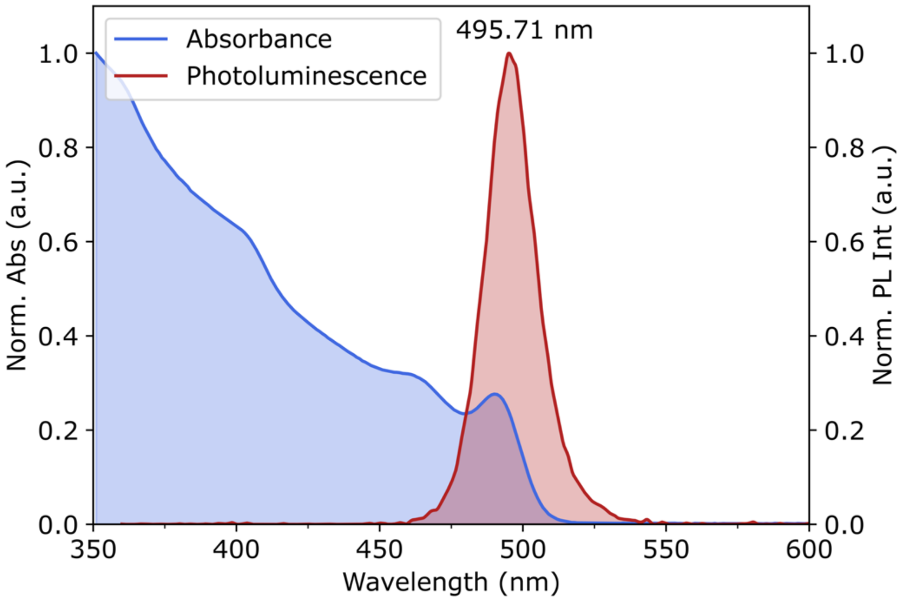

Figure S7: Normalized absorbance and normalized photoluminescence spectrum of quantum confined $CsPbBr_3$ NCs in hexane (5 µL in 3 mL hexane). The excitation wavelength λ_{ex} was set to 350 nm. The maximum of the PL spectrum can be found at 495.71 nm with an FWHM of the PL peak of 21.24 nm.

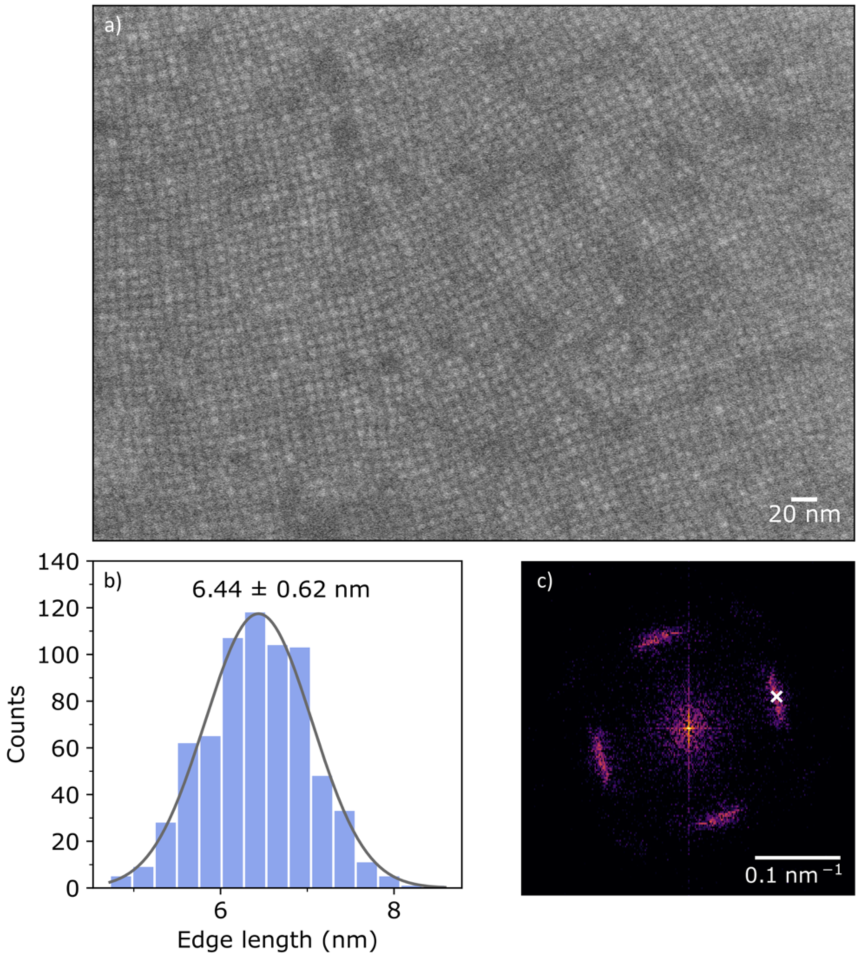

Figure S8: a) SEM image of a thin film of quantum confined $CsPbBr_3$ NCs deposited on a silicon wafer by spin coating. b) Histogram of the size distribution of the NC resulting from the SEM image. The gray curve represents a Gaussian fit from which the size of the NC of 6.44 ± 0.62 nm can be deducted. c) FFT power spectrum of the SEM image. The white cross marks the position corresponding to the average center-to-center distance of 8.44 nm.

Table S1: Size distributions of the NCs in solution obtained by counting and FFT for the different compositions used in the crystallizations.

	$CsPbBr_3$	$CsPbBr_2Cl$	$CsPbCl_3$	smaller $CsPbBr_3$
Edge length (nm)	7.45 ± 0.89	8.38 ± 1.35	7.87 ± 1.15	6.44 ± 0.62
FFT (nm)	11.41 ± 2.13	12.17	12.43	8.44

Section S2: Properties of the $CsPbX_3$ nanocrystals in the supercrystals

This section provides supplementary SEM images showing the surface of the SCs after the different crystallization methods used, as well as a comparison of the vertical expansion of the SCs obtained by our two-layer diffusion technique and the evaporation technique.

S2.1 – Analysis of NC size in SCs, interparticle distance in SCs, and the dimensions of SCs.

The surface of a $CsPbBr_2Cl$ SC using the former method is shown in **Fig. S9** along with the FFT power spectrum of this area. The FFT power spectrum demonstrates a high homogeneity of the arrangement and size of the NCs within the SC by an extremely refined four-fold symmetry with an average center-to-center distance of 19.36 nm, which is consistent with the results from the main text.

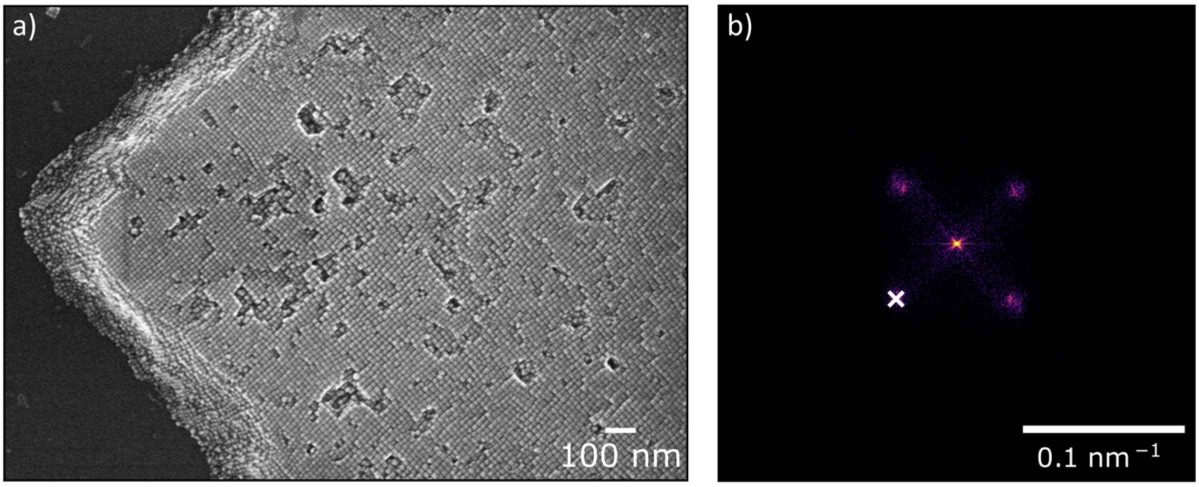

Figure S9: a) SEM image of a corner of a $CsPbBr_2Cl$ SC after crystallization with our two-layer phase diffusion technique showing individual NCs. b) FFT power spectrum of the SEM image depicts extremely refined four-fold symmetry simultaneously demonstrating high homogeneity of the arrangement and the size of the NCs. Position of average center-to-center distance in the SC is marked at 19.36 nm.

As can be seen from **Fig. S10a**, the surface of a $CsPbBr_3$ SC using the evaporation method also exhibits an extremely high periodic order of the individual NCs. In contrast to the two-layer diffusion technique, fewer defects are visible on the SC surface. The FFT power spectrum for this SC shows again a fourfold symmetrical arrangement of very sharp signals with an average center-to-center distance of 9.09 nm.

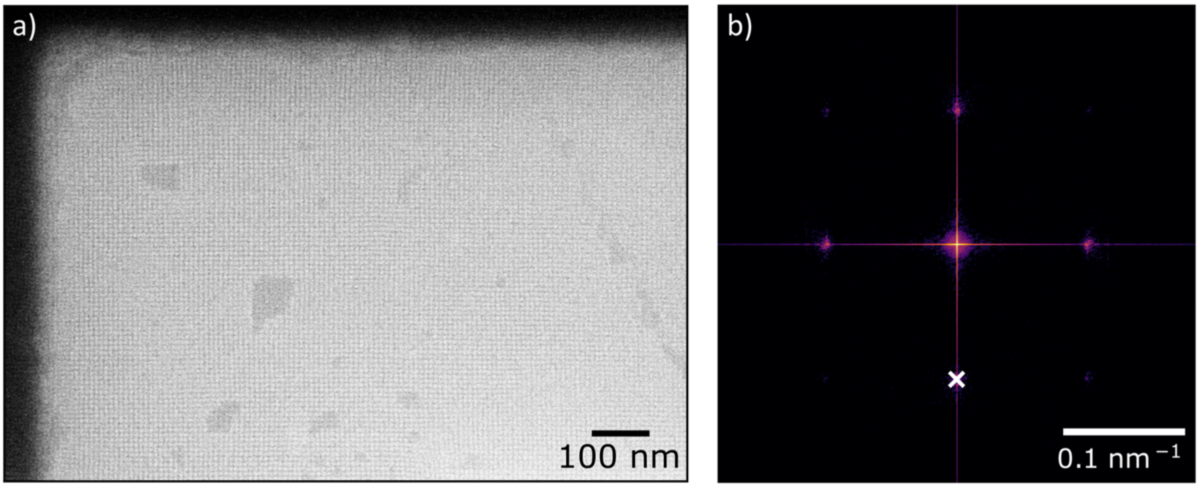

Figure S10: a) SEM image of a corner of $CsPbBr_3$ SC after crystallization from toluene by slow evaporation showing individual NCs. b) FFT power spectrum of the SEM image depicts extremely refined four-fold symmetry simultaneously demonstrating high homogeneity of the arrangement and the size of the NCs. Position of center-to-center distance in the SC is marked at 9.09 nm.

Fig. S11 shows SEM images of a $CsPbBr_2Cl$ SC obtained from a classical diffusion-based crystallization technique in which a particle solution in toluene was overlaid with the same amount of ethanol. From the magnification of the upper right corner of the SC, it can be shown that no individual NCs can be resolved on the surface of the SC. This crystallization technique was therefore not pursued any further.

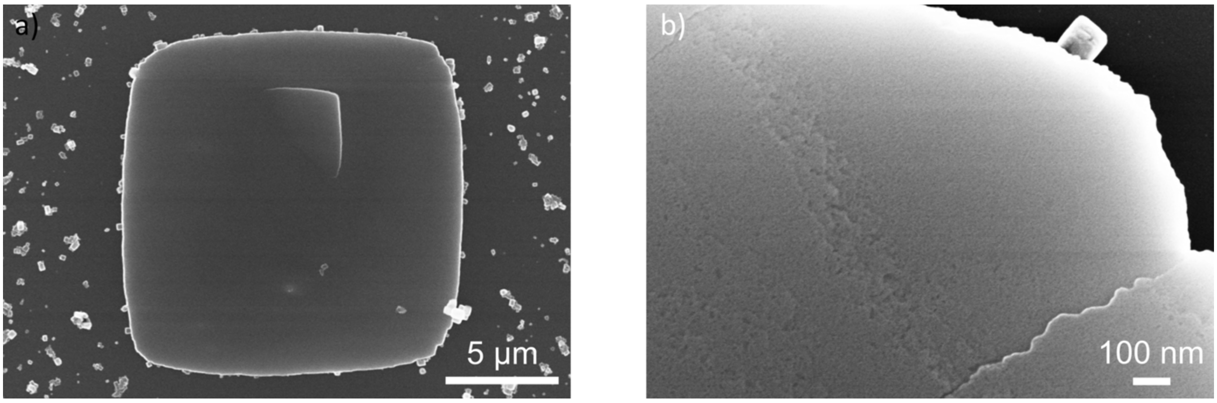

Figure S11: a) SEM image of a $CsPbBr_2Cl$ SC after crystallization from toluene with ethanol. b) magnification of the upper right corner of the $CsPbBr_2Cl$ SC revealing a featureless surface of the SC without any individual distinguishable NCs.

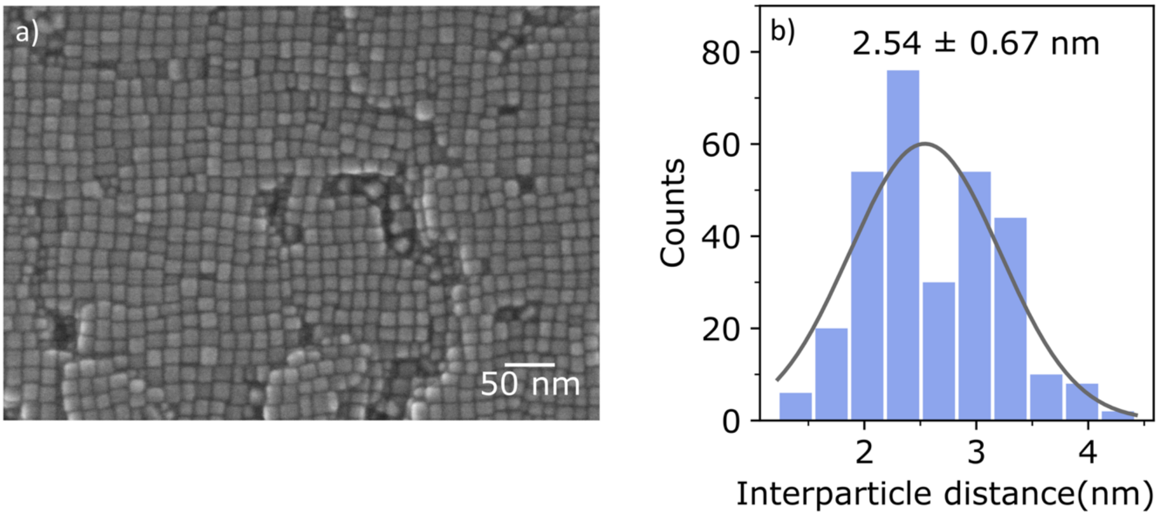

Figure S12: a) Magnified SEM image of the surface of a $CsPbBr_2Cl$ SC. b) Histogram of the interparticle distance derived from the SEM image in a). The gray curve represents a Gaussian fit from which the interparticle distance between adjacent NCs of 2.54 ± 0.67 nm can be deducted.

From the SEM image in **Fig. S12**, showing a magnification of the surface of a $CsPbBr_2Cl$ SC, we measured the interparticle distance between adjacent NCs to derive a value for the size of the ligand shell around the NCs. The resulting interparticle distance distribution is displayed in **Fig.12b** with a Gaussian function fitted to the distribution. By counting over 100 interparticle distances we obtain a mean value of 2.54 nm with a standard deviation of 0.67 nm.

Fig. S13 provides a comparison of the vertical growth of the SCs depending on the crystallization method. Taking the tilt correction into account, the vertical extension of the SCs in **Fig. S13a**, which are obtained using the two-layer diffusion technique is up to 12.4 µm. This corresponds to the largest crystals that could be found using this method. Whereas the SCs grown by the evaporation technique (**Fig. S13b**) only reach a vertical extension of up to 5.1 µm at best. The SEM images in **Fig. S13c-d**, present SCs for both crystallization techniques, that are predominantly formed on the substrates. SCs grown by two-layer phase diffusion typically measure around 2-3 µm in height (**Fig. S13c**), compared to a few hundred nanometers measured in SCs grown by slow-evaporation (**Fig. S13d**). The SCs displayed herein are representative examples that are utilized in AFM measurements.

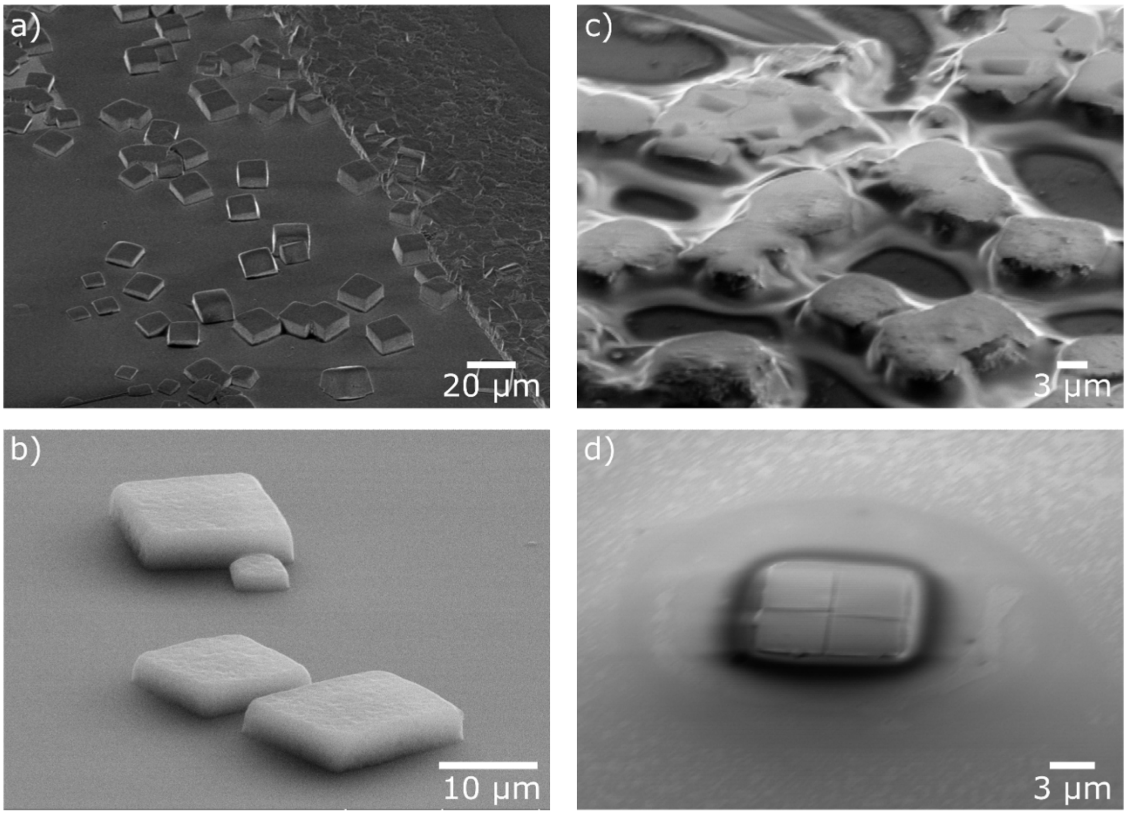

Figure S13: Comparison of vertical growth of SCs from different crystallization methods. a) and b) SEM images of SCs consisting of $CsPbBr_3$ NCs under a viewing angle of 54° on a glass substrate and at a viewing angle of 70° on a silicon wafer respectively, obtained using the two-layer phase diffusion technique. c) and d) SEM images of SCs consisting of quantum confined $CsPbBr_3$ NCs under a viewing angle of 54° on a silicon wafer obtained by slow evaporation.

S2.2 – Cross-section along the vertical and horizontal axis of a SC

In addition to the increasing SC lattice parameter towards the center of the SCs, we investigated the size of the NCs along the vertical axis of the SC. Therefore, a $CsPbBr_3$ SC was intentionally broken using the microgrippers and placed on a silicon wafer that was mounted on a SEM sample holder with an inclination of 45°. Under a viewing angle of 45°, the breaking edge can be aligned orthogonally to the electron beam. The broken SC is shown in **Fig. S14** together with high resolution images of the cross-section along the breaking edge. The SEM images were analyzed by FFT with the obtained FFT power spectra next to the corresponding SEM image (**Fig. S15**). The distance from the zero-frequency point is marked with the equivalent average center-to-center distance. All values obtained along the cross-section are within the resolution limit of the SEM, thus we assume no significant growth of the NCs in the vertical axis.

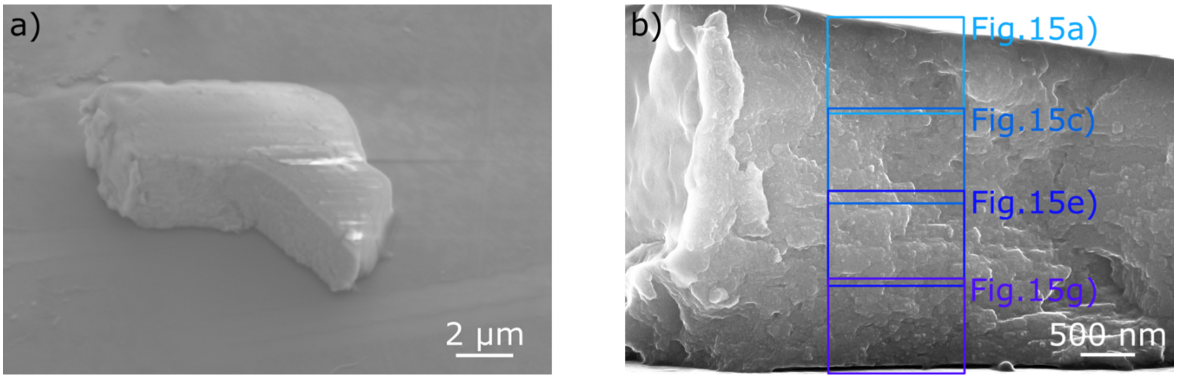

Figure S14: a) SEM overview image of an intentionally broken $CsPbBr_3$ SC mounted on a tilted sample holder (45°). b) Magnification of the breaking edge under a viewing angle of 45°. Areas with further magnification of a representative cross-section along the breaking edge are marked.

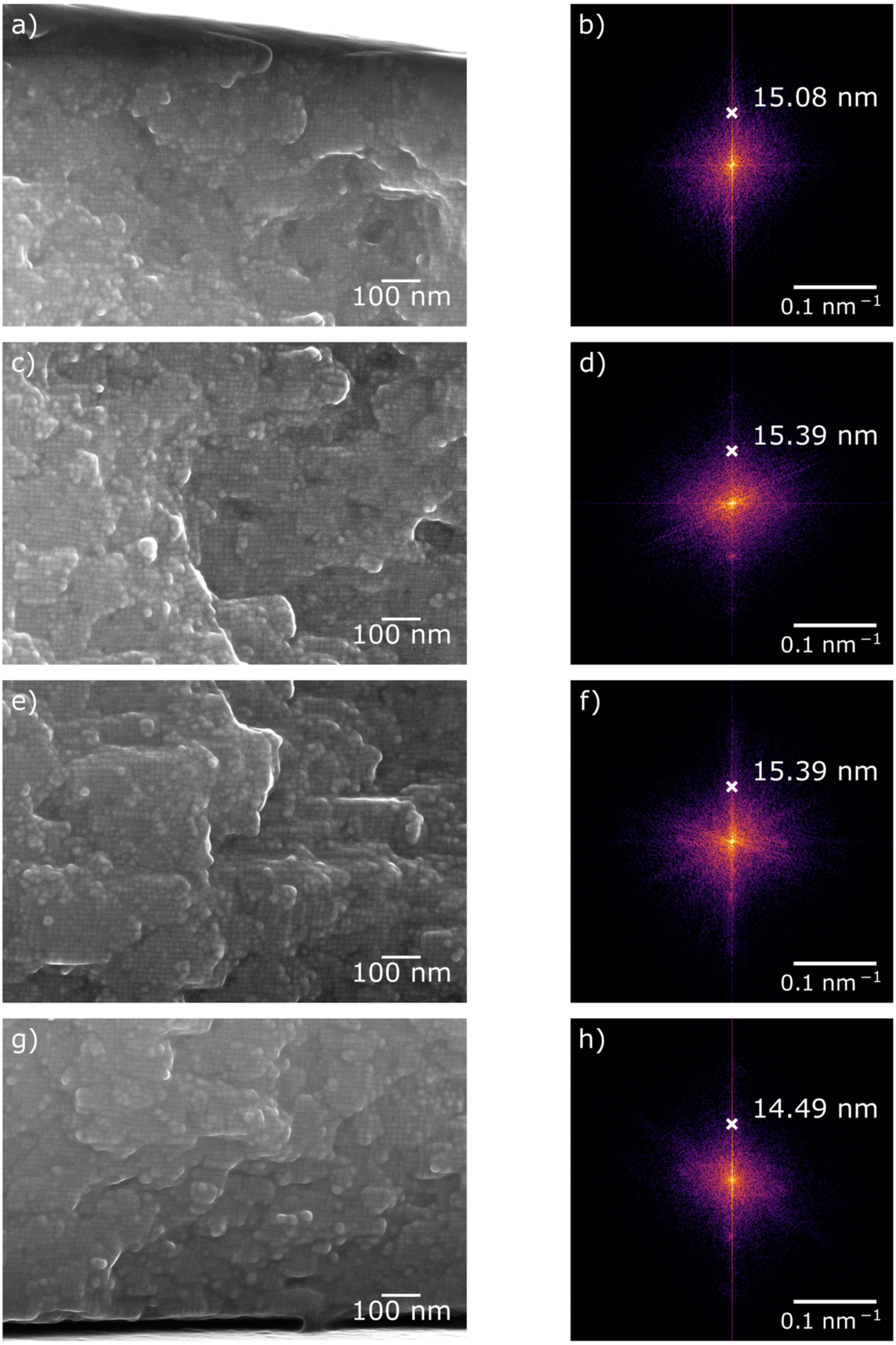

Figure S15: Magnified SEM images of the areas along the cross-section marked in Fig. S14b with the corresponding FFT power spectra. Positions of enhanced intensity are marked and associated center-to-center distances are indicated.

In addition to measuring the size of the NC along a vertical axis of the breaking edge, we determined the size of the NCs along the horizontal axis inside the SC resulting from the breaking edge. The regions that were used for these measurements are shown in **Fig. S16** together with the FFT power spectra obtained for these regions. The average center-to-center distances between adjacent NCs resulting from the FFT power spectra ascend from 11.91 nm for the edge region to 13.72 nm for the intermediate region to 16.66 nm for the region close to the center of the SC. This tendency is consistent with the results from the observations of the remaining monolayer after lifting a SC in the main text (**Fig. 4**).

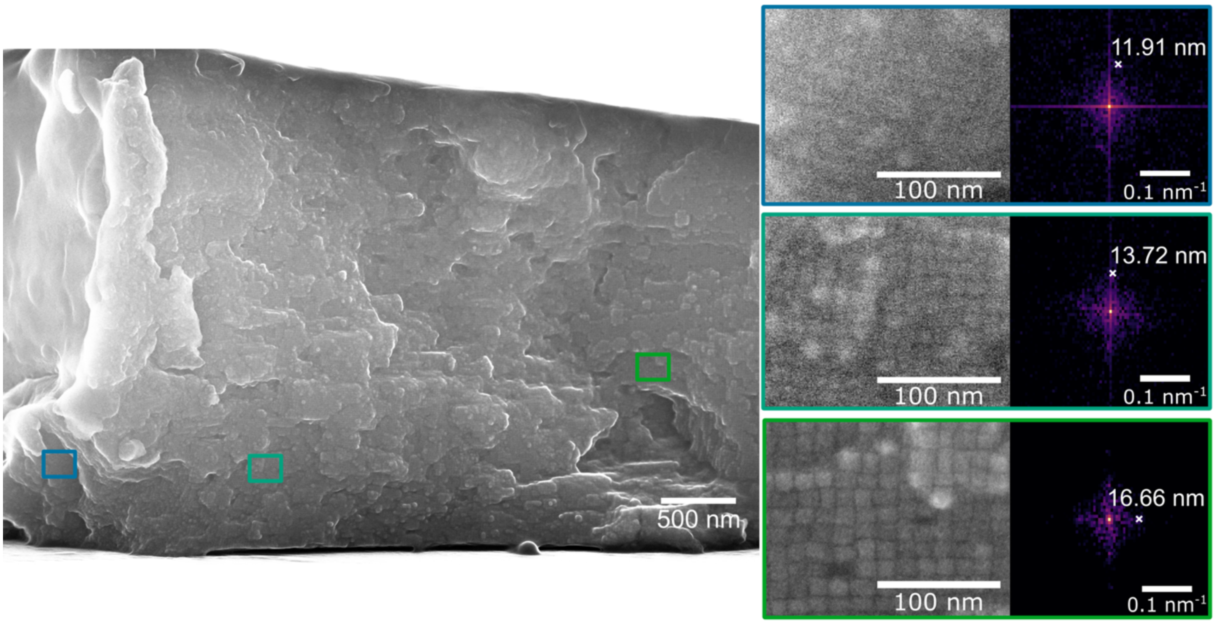

Figure S16: Magnified SEM image of the breaking edge of the SC displayed in ***Fig. S14a*** *under a viewing angle of 45°. Areas with further magnification along the horizontal direction are marked and depicted on the right side of the figure with the corresponding FFT power spectra. Positions of enhanced intensity are marked and associated center-to-center distances are indicated.*

Section S3: X-ray nanodiffraction data analysis

The following section intends to present the workflow used to evaluate the X-ray nanodiffraction data. The $CsPbBr_3$ SC displayed in **Fig.2** and **Fig.3** of the main text is used to exemplify the process.

S3.1 – Processing raw data

The incident X-ray beam is linearly (horizontally) polarized. To account for the effect on the scattering intensity a polarization matrix $P(x, y)$ with the size of the detector in pixels is calculated according to eq. S1, where 2θ is the scattering angle and φ is the azimuthal position of each pixel relative to the position of the direct beam on the detector:

$$P(x, y) = (\sin(2\theta) \cdot \sin(\varphi))^2 + \cos(2\theta)^2 \,. \qquad (S1)$$

The azimuthal position φ for each pixel (x,y) relative to the direct beam (X_{beam}, Y_{beam}) is calculated according to eq. S2:

$$\varphi = \arctan\left(\frac{y - Y_{beam}}{x - X_{beam}}\right). \qquad (S2)$$

The value of the scattering angle 2θ for each pixel is calculated according to eq. S3, where s_{px} is the pixel size (75 µm) and L is the distance between the sample and the detector ($398 \cdot 10^3$ µm):

$$2\theta = \arctan\left(\frac{s_{px} \cdot \sqrt{(x - X_{beam})^2 + (y - Y_{beam})^2}}{L}\right). \qquad (S3)$$

The resulting polarization matrix is displayed in **Fig. S17**. Each diffraction pattern was corrected for X-ray polarization by multiplication with the polarization matrix.

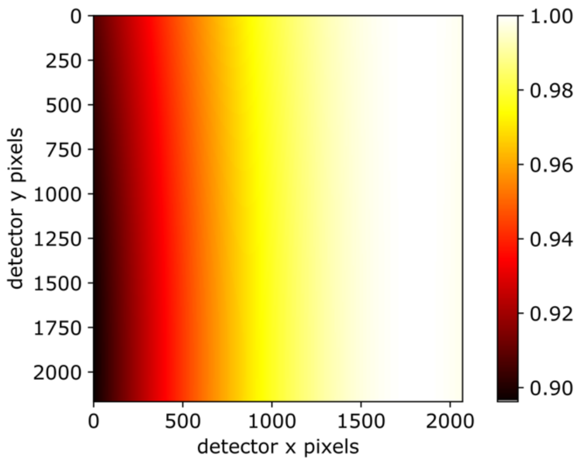

Figure S17: Polarization matrix $P(x, y)$ displayed as a heatmap, where each pixel represents the polarization correction factor calculated for every pixel on the detector.

The raw data recorded by the detector contains artifacts, so-called “hot pixels” and detector gaps between the sensitive panels of the 2D detector, that must be handled prior to analysis. In raw images, the “hot pixels” and the gaps have a value of 2^{32}-1=4294967295, which is the maximum value for a 32-bit integer. Additionally, the beamstop and beamstop holder shadow parts of the detector, resulting in regions of low (but non-zero) intensity. **Fig. S18** illustrates these artifacts in the raw data and demonstrates how a mask is applied to correct them.

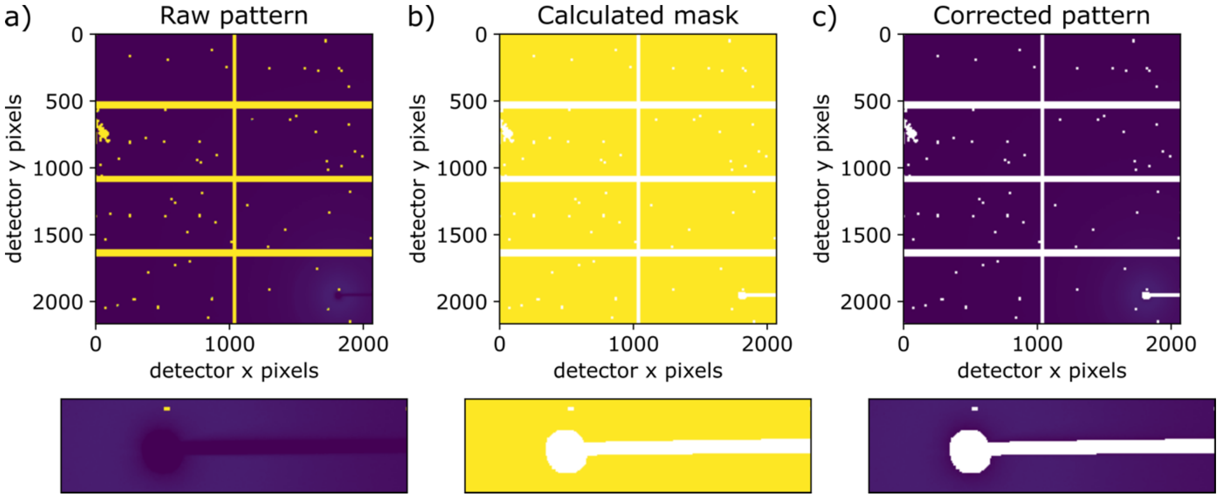

Figure S18: a) Unprocessed background diffraction pattern recorded from the empty Kapton substrate. The inset below shows a magnified view of the beamstop region in the lower right part of the detector. b) Binary mask generated to account for detector artifacts (hot pixels and detector gaps) and the region shadowed by the beamstop. c) Corrected diffraction pattern obtained after applying the mask to the raw data.

The raw diffraction pattern displayed in **Fig. S18a** was recorded from the empty Kapton substrate and is used to generate a binary mask. Within the mask, all pixel values exactly equal to 2^{32}-1 are set to NAN (not a number), accounting for the “hot pixels” and detector gaps. Additionally, all pixel values in the region containing the beamstop and its holder that fall below a defined intensity threshold (in this case 15 counts) are also set to NAN. All remaining pixels in the mask are assigned a value of 1. The resulting binary mask, shown in **Fig. 18b**, is then applied to each individual pattern by multiplication. The corrected background pattern recorded from the Kapton substrate, displayed in **Fig. 18c**, is then subtracted from all individual patterns. This procedure effectively removes artifacts and provides background-corrected patterns for further analysis.

S3.2 – Transforming patterns to polar coordinates

To analyze the diffraction data, the recorded patterns are transformed from the Cartesian coordinate system of the detector to polar coordinates using linear interpolation. In polar coordinates, the data is described in terms of the radial distance q and the azimuthal angle φ with respect to the position of the direct beam. Instead of the real space distance, the magnitude of the scattering vector q is used as the radial coordinate in the polar coordinate system as it aligns the analysis with the reciprocal nature of the diffraction experiment. The process of calculating q from the real space distances on the detector is explained in the following section. A sketch of the geometry of the nanodiffraction experiments is displayed in **Fig. S19**.

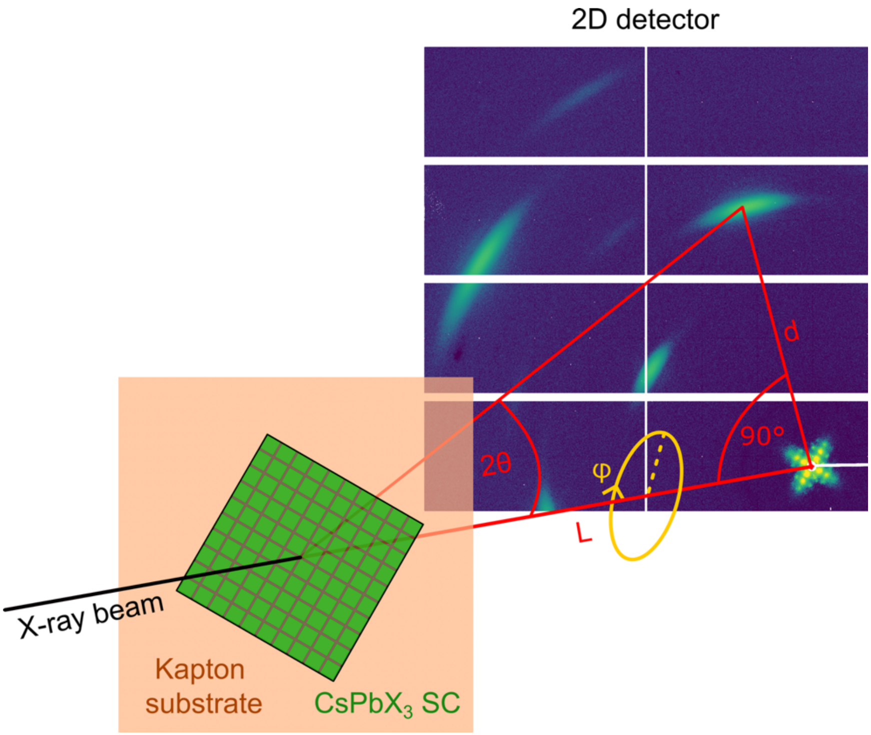

Figure S19: Sketch of the X-ray nanodiffraction experiment in transmission geometry. The X-ray beam passes through the $CsPbX_3$ SC and a diffraction pattern is recorded on the 2D detector located behind the sample at a distance of L. The real space distance d from the transmitted beam position on the beamstop to any point on the detector is related to the Bragg angle θ, while the azimuthal angle φ is defined with respect to the transmitted beam.

The general form of Bragg's law, which relates the scattering angle θ to the wavelength λ and the lattice spacing d_{hkl} is:

$$n\lambda = 2d_{hkl}\sin(\theta). \qquad (S4)$$

The magnitude of the scattering vector q is directly related to the scattering angle as

$$q = \frac{4\pi}{\lambda}\sin(\theta). \qquad (S5)$$

The scattering angle 2θ of any point on the detector can be calculated from the real space distance d from that point to the transmitted beam position and the detector-to-sample distance L:

$$\frac{d}{L} = \tan(2\theta). \qquad (S6)$$

Combining the two equations above gives

$$q = \frac{4\pi}{\lambda}\sin\left(\frac{1}{2}\arctan\left(\frac{d}{L}\right)\right). \qquad (S7)$$

Fig. S20 shows an exemplary diffraction pattern recorded from the $CsPbBr_3$ SC both in Cartesian and in polar coordinates. In this work, polar plots are displayed as $I(q,\varphi)$ plots.

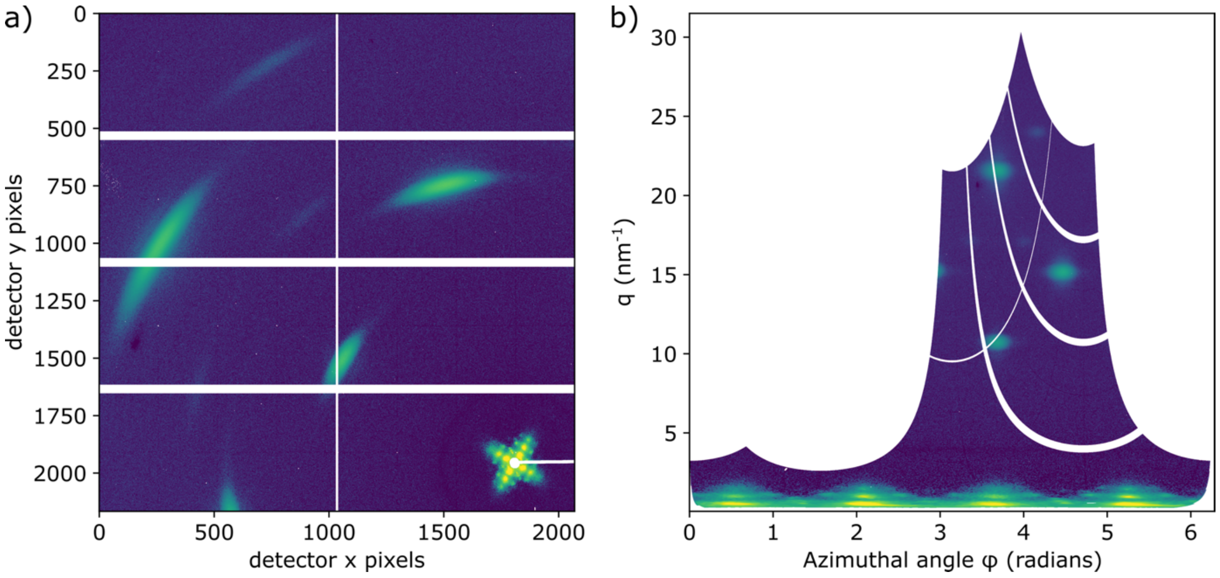

Figure S20: An exemplary diffraction pattern recorded from the $CsPbBr_3$ SC in a) Cartesian coordinates and b) polar coordinates displayed as an $I(q,\varphi)$ plot.

S3.3 – Generating spatially-resolved maps of the sample

Scattering intensity maps of the sample are generated by integrating regions of interest in the averaged radial profiles of the diffraction patterns. For an exemplary diffraction pattern, the small- and wide angle X-ray scattering (SAXS and WAXS) regions in polar coordinates, as well as their radial profiles, are displayed in **Fig. S21**.

The SAXS intensity maps of the samples in this work were generated by integrating the radial profile for $q < 2\ \mathrm{nm}^{-1}$(see **Fig. S21d**). The WAXS intensity maps were generated as the sum of the integrated radial profile around the 100_{AL}, 110_{AL} and 200_{AL} peaks in a range of $\pm 1\ \mathrm{nm}^{-1}$ (see **Fig. S21e**).

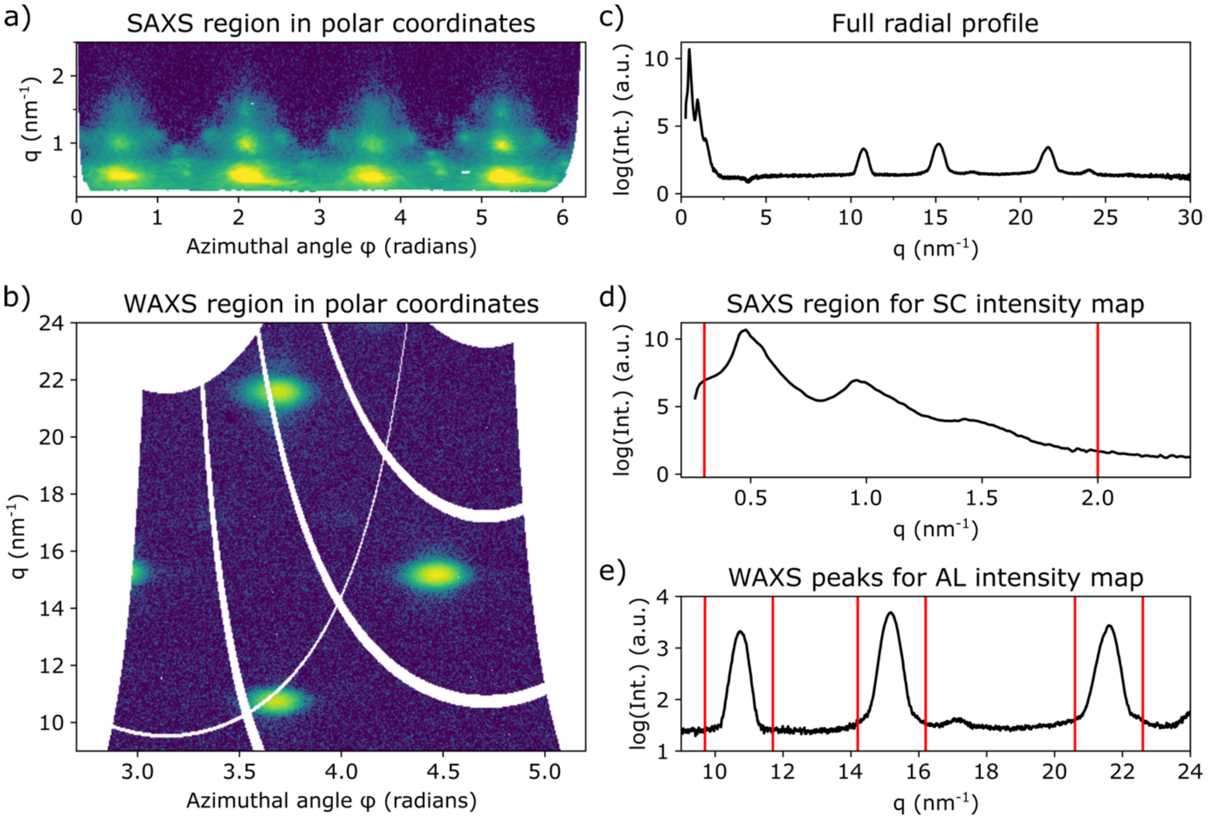

Figure S21: Exemplary diffraction pattern recorded from the $CsPbBr_3$ SC in polar coordinates. a) displays the SAXS region. b) the WAXS region. c) The full radial profile of the diffraction pattern. d) Radial profile of the SAXS intensity, where the area to be integrated for the intensity map is indicated. e) Radial profile of the WAXS region, where the three areas are indicated.

S3.4 – Obtaining the average diffraction pattern of the sample

The next step of the analysis is calculating the spatially averaged diffraction pattern of the $CsPbBr_3$ SC. Before averaging, diffraction patterns collected from the bare Kapton substrate around the SC are excluded. The separation of patterns from the Kapton substrate and the SC is achieved using binary intensity thresholding, as illustrated in **Fig. S22**.

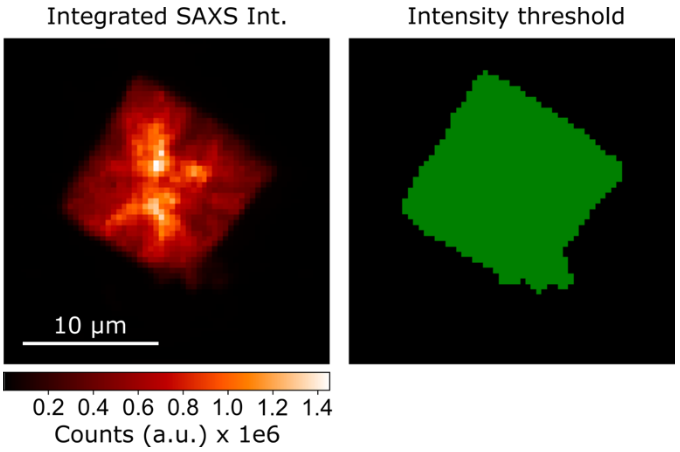

Figure S22: Using binary intensity thresholding of the intensity map to separate spatial locations of the scanned sample area that belong to either the SC (green) or regions outside of the SC (black)

In principle, either the SAXS or WAXS intensity map can be used for the thresholding to distinguish between the position of an X-ray beam on SC sample and on the bare Kapton substrate. In this case, the positions, for which the corresponding diffraction patterns have the integrated SAXS intensity values >15% of the maximum integrated intensity, were classified as belonging to the SC. The integration was performed over the whole SAXS region for $q < 2\ \mathrm{nm}^{-1}$. Patterns classified as belonging to the bare Kapton substrate were excluded from further analysis. This significantly reduces computational effort in the subsequent fitting process. The spatially averaged diffraction patterns of the $CsPbBr_3$ SC is displayed in **Fig. S23**.

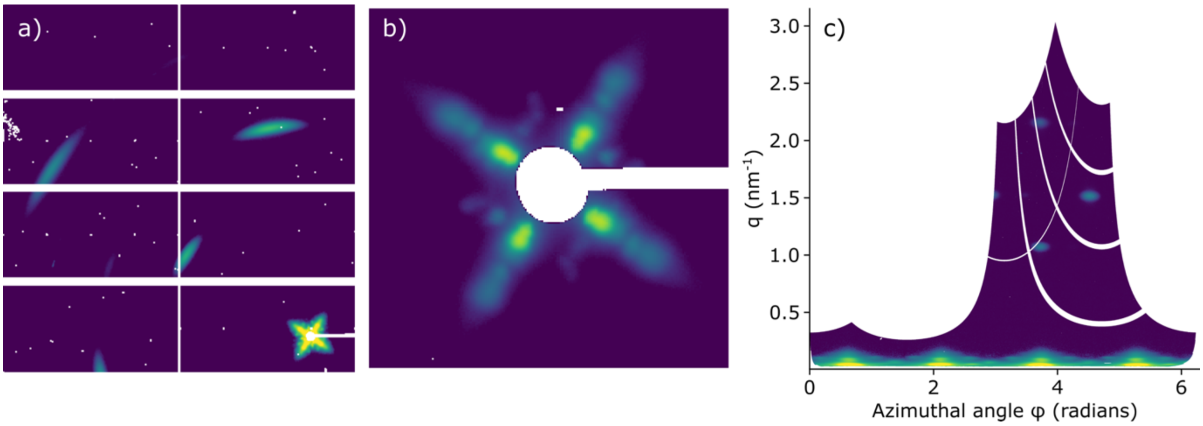

Figure S23: Spatially averaged diffraction pattern of the $CsPbBr_3$ SC in a logarithmic color scale. a) The full spatially averaged pattern in cartesian coordinates. b) The SAXS region of the spatially averaged pattern in cartesian coordinates. c) $I(q,\varphi)$ plot of the full spatially averaged pattern in polar coordinates.

S3.5 – Selecting individual peaks for fitting from the average diffraction pattern

We extract the maximum value of the four first order SAXS peaks in the radial direction (two peaks corresponding to 100_{SL} and 010_{SL} reflections, respectively). For this sample, as indicated by the red line in **Figs. S24a** and **S24b**, this value is at $q = 0.41\ \mathrm{nm}^{-1}$. From the azimuthal profile at this radial position, displayed in **Fig. S24c**, we extract the azimuthal positions of the peak maxima. Here, the azimuthal angles of the first order SAXS peaks in radians are at φ = 0.597 (100_{SL} Peak1), φ = 2.167 (010_{SL} Peak2), φ = 3.738 ($\bar{1}00_{SL}$ Peak3), and φ = 5.310 ($0\bar{1}0_{SL}$ Peak4). We generate four arrays, each containing one of the first order SAXS peaks, with an azimuthal range of $\Delta\varphi = \pm 0.436$ radians (25 degrees) and a radial range of $\Delta q = 0.41\ \mathrm{nm}^{-1}$ around each peak. These arrays are displayed in **Fig. S25**.

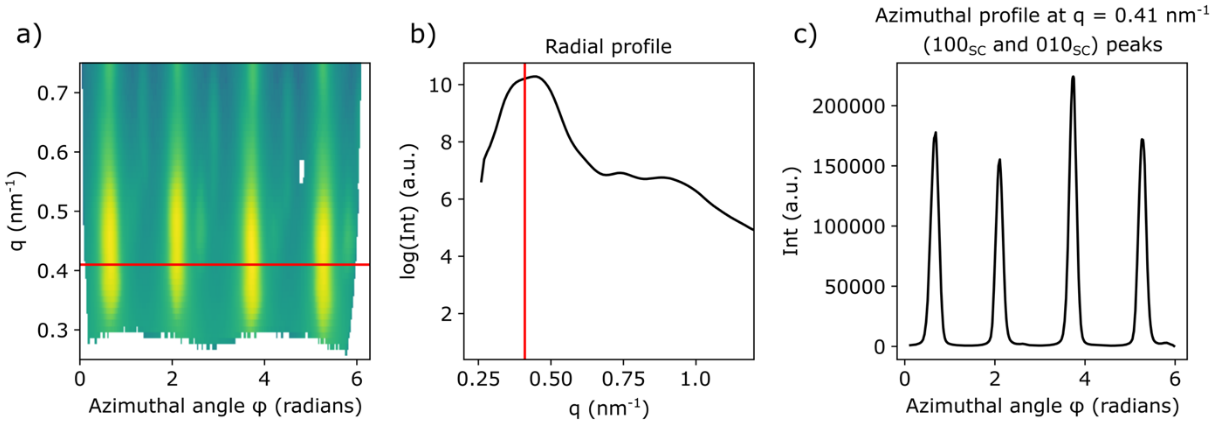

Figure S24: Selecting the radial and azimuthal positions of the first order SAXS peaks (100_{SC} and 010_{SC}) in the spatially averaged diffraction pattern. a) The SAXS region of the average diffraction pattern in polar coordinates (logarithmic intensity scaling). b) The radial profile of the SAXS region. c) The azimuthal profile at the radial peak maximum of the first order SAXS peaks.

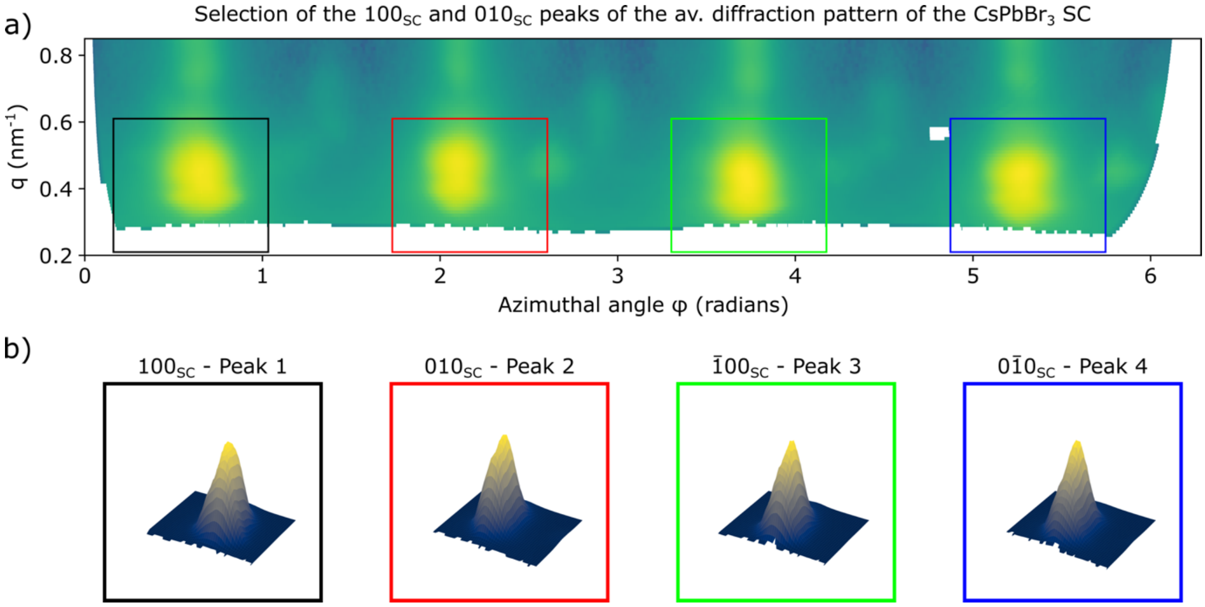

Figure S25: a) The extent of the four arrays, each containing one of the four first order SAXS peaks, displayed within the spatially averaged diffraction pattern (logarithmic intensity scaling). b) A 3D representation of the individual Bragg peaks contained in the arrays.

By visual inspection of **Fig. S25**, we can confirm that the ranges $\Delta\varphi$ and Δq were chosen sufficiently large so that the arrays for the first order SAXS peaks contain the full peaks and some background, which is important for accurate fitting of the data. As these arrays contain the entire spatially averaged peaks, they also contain the peaks of the individual diffraction patterns, that make up the spatially averaged pattern. Therefore, these arrays can be employed to extract the first order SAXS peaks at all positions of the sample. An analogous procedure is applied to obtain arrays containing the atomic lattice peaks in the WAXS region of the average diffraction pattern.

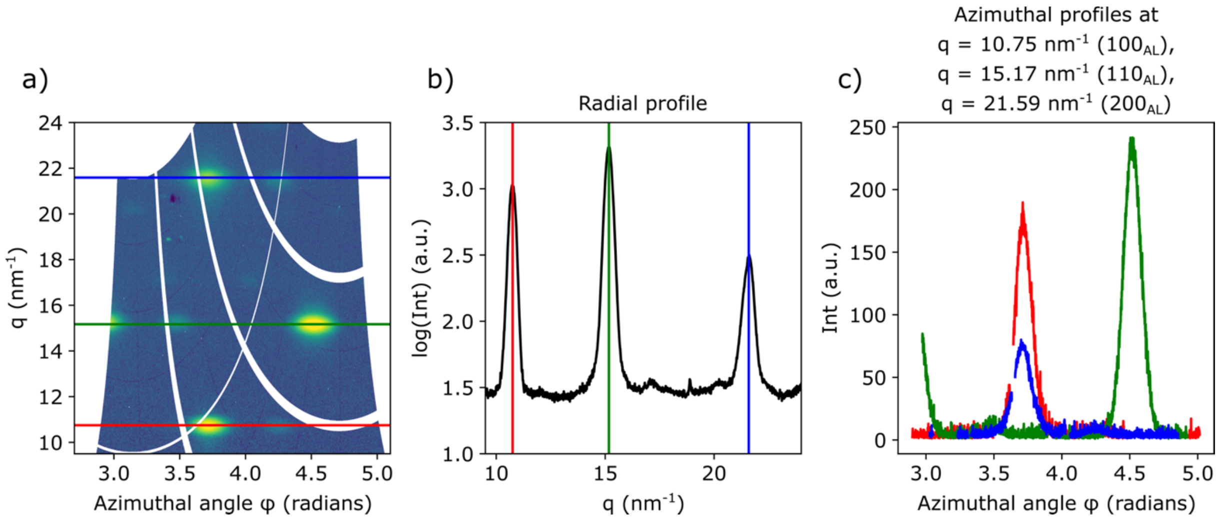

Figure S26: Selecting the radial and azimuthal positions of the three atomic lattice peaks (100_{AL}, 110_{AL}, and 200_{AL}) in the average diffraction pattern. a) The WAXS region of the average diffraction pattern in polar coordinates (logarithmic intensity scaling). b) The radial profile of the WAXS region. c) The azimuthal profiles at the radial peak maxima of the three atomic lattice peaks.

The radial positions of the atomic lattice peaks, as indicated in **Figs. S26a** and **S26b**, are at $q = 10.75\ \mathrm{nm}^{-1}$ (100_{AL}), $q = 15.17\ \mathrm{nm}^{-1}$ (110_{AL}), and $q = 21.59\ \mathrm{nm}^{-1}$ (200_{AL}). From the azimuthal profiles at these radial positions, displayed in **Fig. S26c**, we extract the azimuthal positions of the peak maxima. For this sample, the azimuthal angles of the atomic lattice peaks are at φ = 3.712 (100_{AL}), φ = 4.520 (110_{AL}), and φ = 3.700 (200_{AL}). For the atomic lattice peaks, we define three arrays with an azimuthal range of $\Delta\varphi = \pm 0.436$ radians (25 degrees)

and a radial range of $\Delta q = \pm 1\ \mathrm{nm}^{-1}$ around each peak. These arrays are displayed in the following figure **Fig. S27**.

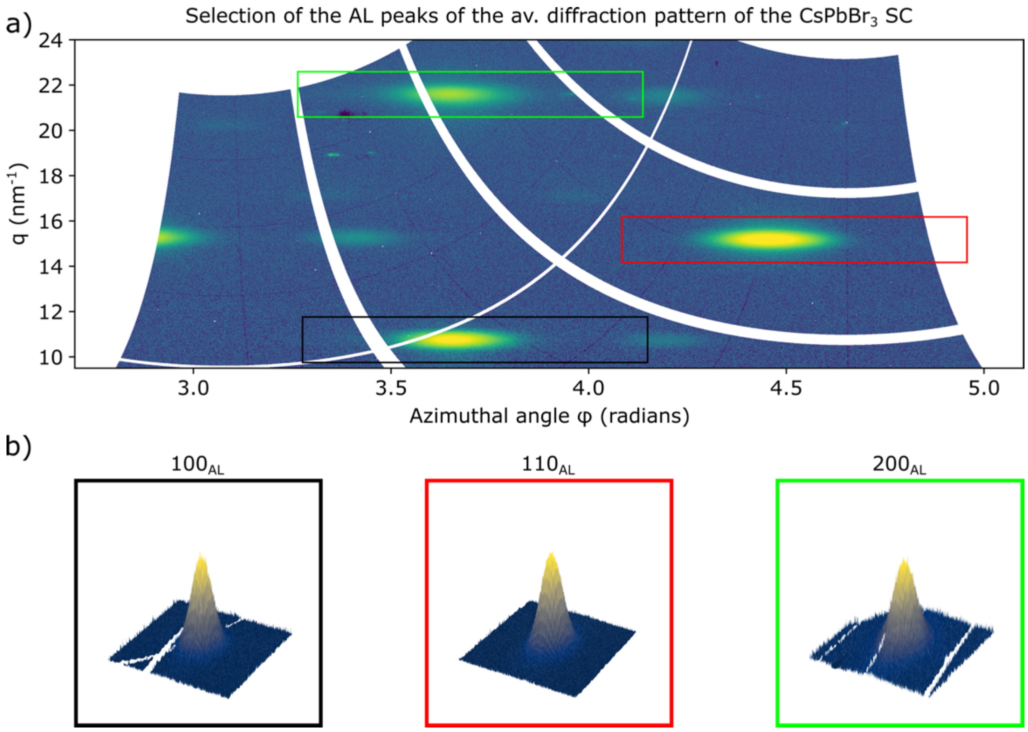

Figure S27: a) The extent of the three arrays, each containing one of the atomic lattice Bragg peaks, displayed within the spatially averaged diffraction pattern (logarithmic intensity scaling). b) A 3-dimensional representation of the individual Bragg peaks contained in the arrays.

S3.6 – Fitting the SAXS and WAXS Bragg peaks

2D Gaussians are used to fit the individual SAXS and WAXS peaks in the arrays:

$$f(\varphi, q) = A\ e^{\frac{(\varphi-\varphi_0)^2}{2\sigma_\varphi^2} + \frac{(q-q_0)^2}{2\sigma_q^2}} + B \qquad (S8)$$

The following parameters are fitted: The amplitude of the peak A, the background level B, the azimuthal peak position φ_0, the radial peak position q_0, the azimuthal width σ_φ, the radial width σ_q.

We use the average diffraction pattern of a SC to check the quality of the 2D-Gaussian fits employed for fitting the individual peaks. This is exemplified in **Fig. S28**, where the fit of the average 100_{AL} peak from **Fig. S27b** is displayed.

The fitted parameters are used to calculate structural properties of the sample in the following step.

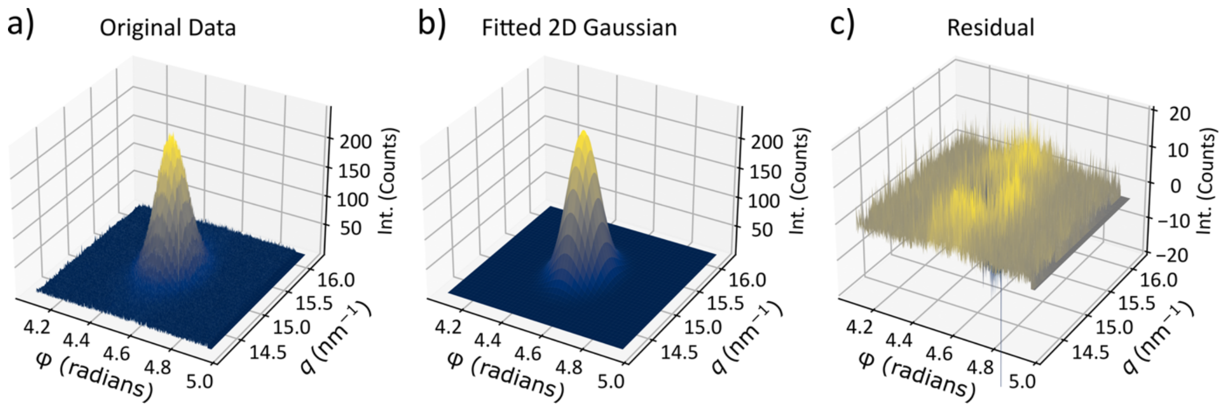

Figure S28: Exemplary 2D Gaussian fitting of the 110_{AL} peak of the average diffraction pattern recorded from a $CsPbBr_3$ SC. a) displays the original data, b) the 2D Gaussian fit, and c) the residual.

S3.7 – Calculating structural properties of NCs and SCs from the diffraction peaks

The angle γ between the unit vectors of the SC unit cell is calculated as

$$\gamma = \frac{1}{2}\left(\varphi_0^{100\text{SC}} + \varphi_0^{\bar{1}00\text{SC}} - \pi\right) - \frac{1}{2}\left(\varphi_0^{010\text{SC}} + \varphi_0^{0\bar{1}0\text{SC}} - \pi\right). \qquad (S8)$$

The orientation ψ of the SC lattice is calculated as

$$\psi = \frac{\frac{1}{2}\left(\varphi_0^{100\text{SC}} + \varphi_0^{\bar{1}00\text{SC}} - \pi\right) + \frac{1}{2}\left(\varphi_0^{010\text{SC}} + \varphi_0^{0\bar{1}0\text{SC}} - \pi\right)}{2}. \qquad (S10)$$

The average q-position of the first order SAXS peaks $\langle q_0 \rangle$ was calculated as the mean value of the four equivalent SAXS peaks:

$$\langle q_0 \rangle = \frac{1}{4}\left(q_0^{100\text{SC}} + q_0^{010\text{SC}} + q_0^{\bar{1}00\text{SC}} + q_0^{0\bar{1}0\text{SC}}\right) \qquad (S11)$$

The average center-to-center distance between neighboring NCs in the SC (the lattice parameter of the SC) $\langle a_{SC} \rangle$ is calculated as

$$\langle a_{SC} \rangle = \frac{2\pi}{\langle q_0 \rangle \sin\gamma}. \qquad (S12)$$

The pseudo-cubic atomic lattice parameter of the $CsPbX_3$ NCs a_{AL} is calculated as

$$a_{\text{AL}} = \frac{2\pi}{q_0^{100_{\text{AL}}}}. \qquad (S13)$$

Section S4: Atomic force microscopy

S4.1 – Determination of the Young modulus

Each pixel of the mechanical mapping shown in **Fig. 6** of the main text represents the value of the Young modulus that was measured at a specific position. These values were obtained by fitting single force curves at each spatial position. Every single force curve was obtained as an average of a retraction and approach curves. The Nanoscope 9.2 Software was used for measurements and fitting of measured curves. The Nanoscope Analysis 3.0 Software and the Gwyddion software have been utilised for the data analysis.[3] Measurements and calibrations were done in air using the PeakForce QNM mode and calibration standards recommended by the manufacturer. Below we describe the model used to obtain the Young modulus from the force curve.

The most employed model for data processing in AFM nanoindentation is the Hertz model. This model operates under the assumption that the cantilever tip is spherical, and that the indentation depth is significantly smaller than the tip radius. The indenter is non-deformable and no other forces are assumed between the tip and the surface to be indented.[4] In this model, the applied force F (set to 150 nN) is related to the indentation depth δ as

$$F = \frac{4}{3}\frac{E}{(1-v^2)}\sqrt{R}\delta^{\frac{3}{2}}, \qquad (S14)$$

where E represents the Young modulus, v is the Poisson ratio (estimated at 0.3), and R is the tip radius. However, the model does not consider adhesion forces at the contact surface. The adhesion is included in the Hertz-based Derjagin-Muller-Torpov- (DMT) or Johnson-Kendall-Roberts- (JKR) model. DMT extended Hertz's model includes an additional term that takes the adhesion forces into account

$$F_{ad} = 2\pi RW, \qquad (S15)$$

where W describes the work of adhesion. Consequently, the DMT model can be described as[5]

$$F = \frac{4}{3}\frac{E}{(1-v^2)}\sqrt{R}\delta^{\frac{3}{2}} + F_{ad} \qquad (S16)$$

The rationale behind the utilization of the DMT model over the JKR model is elucidated as follows: the JKR model pertains to large tips and soft samples characterised by strong adhesion, while the DMT model is pertinent to small tips and hard samples with weak adhesion. In the context of AFM applications, the radius of the tips employed (RTESPA 75) is small, and the adhesion forces exerted by the SCs are minimal. The calculation of the Young modulus can be performed by fitting a retraction force curve with the Young modulus as the fit parameter.[4–6]

The value of the Young modulus can be calculated from eq. S16, if the values of all other parameters are known. The force F can be defined from Hooke's law, assuming that the oscillating cantilever can be approximated as a harmonic oscillator:

$$F = k \cdot \Delta z. \qquad (S17)$$

Here k is the spring constant of the cantilever and Δz is the cantilever deflection. To obtain sufficient information regarding the indentation depth, an accurate deflection sensitivity is needed.

In the following subsections, we will explain the calibration of the deflection sensitivity, calibration of spring constant k and measurement of the tip radius.

S4.2 – Calibration of the deflection sensitivity

The hard surface contact technique is employed to calibrate the sensitivity of the cantilever by pressing the imaging tip against a surface. The stiffness of the surface is significantly greater than the stiffness of the cantilever. For calibration, five separate force-distance curves were recorded on the SAPPHIRE-12M calibration

standard from Bruker. The deflection sensitivity S is defined as the photodiode voltage V_{dif} divided by the distance travelled by the piezo $\delta_{F\ max}$[7]:

$$S = \frac{V_{dif}}{\delta_{F\ max}}. \quad (S18)$$

This parameter is crucial for plotting a force-distance curve because it facilitates the conversion of the raw photodiode signal [V] to the deflection of the cantilever [nm]. Since there are no lateral movements of the cantilever, only changes in the z-direction are relevant. The quadrupole detector of the AFM can thus be divided into two segments, A and B. In the AFM, only one output of the detector, V_{dif}, is recognised, which includes the signals of both segments A-B. $\delta_{F\ max}$ describes a known deflection of the laser by pressing the cantilever onto the calibration sample. The deflection sensitivity can be determined by the slope of the linear part of a force-distance curve shown in **Fig. S29**.[8]

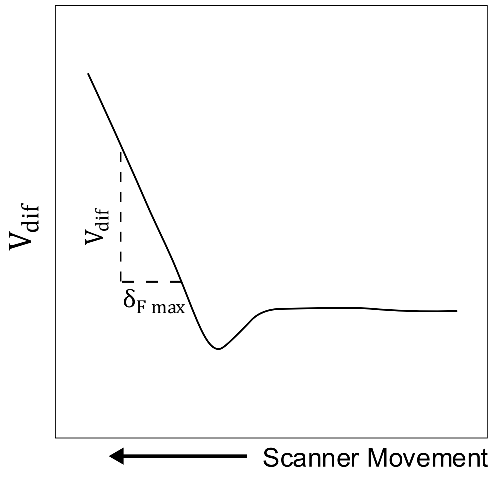

Figure S29: A schematic representation of the sensitivity measurement process, utilizing the force curve of a rigid surface in relation to the stiffness of the cantilever. The linear segment of the curve is employed to determine the deflection sensitivity.

S4.3 – Calibration of the spring constant

The spring constant of a cantilever can be calculated using the Sadder method, which allows a fast and accurate calibration of the spring constant from the geometrical dimensions of a cantilever. The spring constant for a rectangular cantilever is given by

$$k = M_e p_c bhL\omega_{vac}^2 , \quad (S19)$$

where the radial resonant frequency of the cantilever in vacuum is denoted by ω_{vac}, the thickness of the cantilever is h, the width b and the length L. The density of the cantilever is defined as p_c and the normalized effective mass by M_e is the normalized effective mass which takes the value $M_e = 0.2427$ for $L/b > 5$. The application of eq. S19 is limited since the thickness of the coating layer of a cantilever is generally unknown. Sadder et al.[9] proposed a method for calculating the spring constant defining parameters of eq. S19 via measuring the resonant frequency ω_f of the cantilever in a viscous liquid or gas (in our case - air). When the cantilever is placed in air, most damping is caused by viscous drag[10], which results in the quality factor Q_f of the fundamental mode of the cantilever to be much larger than unity, $Q_f \gg 1$. In this case, the vacuum resonant frequency of the cantilever can be estimated as

$$\omega_{vac} = \omega_f \sqrt{1 + \frac{\pi b p_f}{4 p_c h} \Gamma_r(\omega_f)} \quad (S20)$$

and

$$p_c h = \frac{\pi b p_f}{4}\left[Q_f \Gamma_i(c) - \Gamma_r(\omega_f)\right]. \tag{S21}$$

Here p_f is the density of air, and Γ_r and Γ_i are the real and imaginary components of the hydrodynamic function $\Gamma(\omega)$. The function $\Gamma(\omega)$ is independent of the cantilever thickness and density, and depends only on the Reynolds number

$$Re = \frac{p_f \omega b^2}{(4\eta)}, \tag{S22}$$

where η is the viscosity of the fluid (air) surrounding the cantilever.

Inserting eqs. S20, S21 and S22 into eq. S19, one can show that the spring constant can be calculated as

$$k = 0.1906 \cdot p_f b^2 L Q_f \Gamma_i(Re) \omega_f^2 . \tag{S23}$$

The calculation of the spring constant requires knowing the length L and width b of the cantilever, the quality factor Q_f, and the resonant frequency ω_f. The fluid density and fluid viscosity of air are values that are known and equal to 1.18 kg/m^3 and $1.86 \cdot 10^{-5}$kg·s/m, respectively.[11,12] The dimensions of the cantilever have been measured by optical microscopy.

The quality factor Q_f and resonance frequency ω_f have been calculated using the thermal tune function of the Nanoscope Analysis 3.0 software from Bruker. The thermal tune can be explained in accordance with Hutter and Bechhoefer.[13] Since the cantilever is in thermal equilibrium with its surrounding, it fluctuates in response to thermal noise. Assuming that the cantilever is a harmonic oscillator with small damping, in absence of additional sources of noise, especially at the cantilever resonance frequency, the power density of the fluctuations in the cantilever´s displacement has a Lorentzian shape. Given the lack of resonant noise at the cantilever's resonance frequency, the background added by other noise sources can be effectively subtracted as a white noise background, thereby yielding a power spectral density of the thermal fluctuations of the cantilever.

The measurement of the power spectrum was accomplished through the Thermal Tune function within the Nanoscope 9.2 software. By fitting the power spectrum with a Lorentzian line shape, the software can determine the quality factor by dividing the resonant frequency f_0 by the width g of the resonant peak at the half of the maximum power (or at the level of $A_{max}/\sqrt{2}$ from the maximum amplitude A_{max})

$$Q_f = \frac{f_0}{g} . \tag{S24}$$

S4.4 – Measurement of the tip radius

Measurements of the tip radius were accomplished using the ZEISS LEO Gemini 1550 VP electron microscope (**Fig. S30**). A custom-built cantilever holder was made to position the tip with a 90-degree angle to the electron beam.

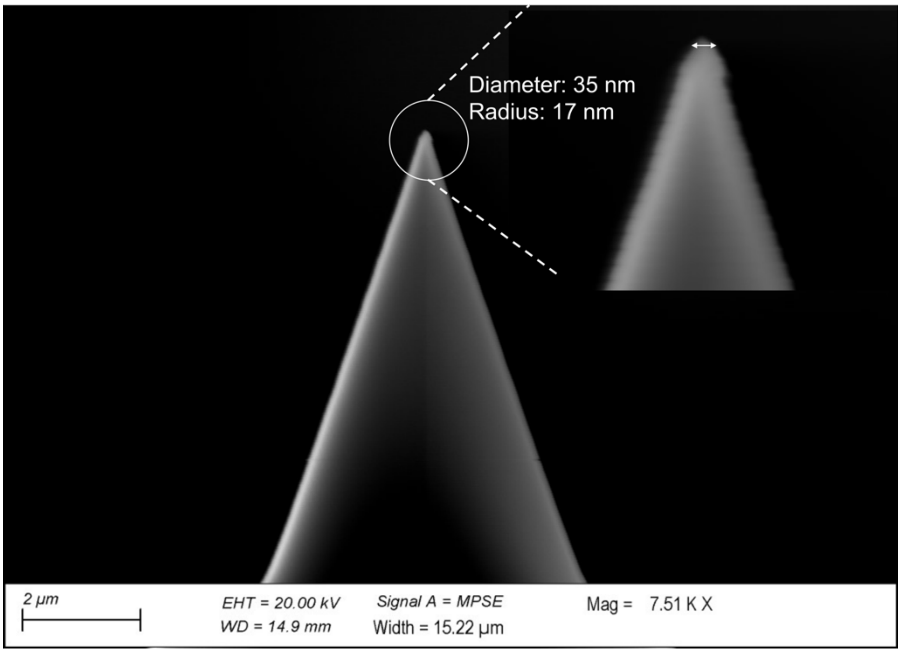

Figure S30: SEM Image of a RTEPSA 75 tip measuring the tip radius. The tip radius was determined as 17 nm.

S4.5 – Statistical representation of the Young moduli of different SCs

Analysis of the Young moduli of different SCs was accomplished using the row/column statistical function feature in Gwyddion. As each pixel represents a given force-distance curve, every pixel can be assigned a value of Young's modulus. By masking the whole SC, as seen in **Fig. S31**, the mean value for the SC can be calculated by adding all Young modulus values and dividing by the total number of pixels in the masked region. In this example, the mean value of the masked region gives a mean value of 3.0 $\pm$ 1.1 GPa (standard deviation).

For a complete overview, the results of 12 different SCs from the two-layer phase diffusion technique and 3 different SCs from the solvent evaporation technique are shown in **Fig. S32**. **Fig. S32a** illustrates the box plot of $CsPbBr_3$ SCs crystallized using the two-layer phase diffusion technique, which have a mean Young modulus value of 3.2 ± 1.2 GPa (standard deviation), with a median of 3.1 GPa. The solvent evaporation SCs shown in **Fig. S32b** have a mean Young modulus of 140.3 ± 67.5 MPa (standard deviation), with a median of 112.3 MPa.

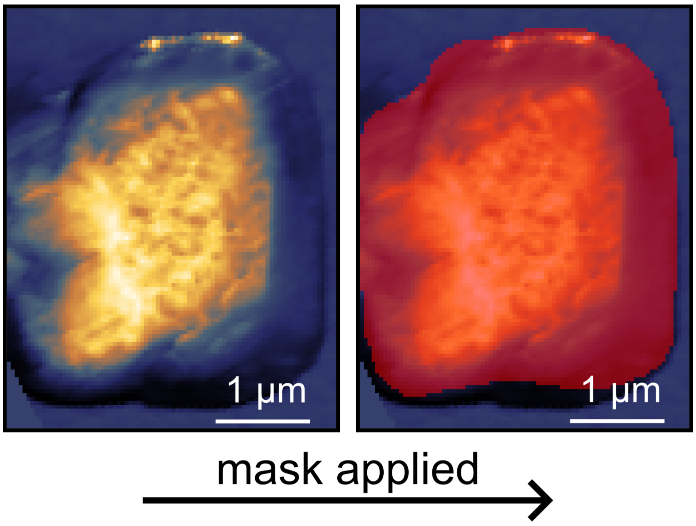

Figure S31: $CsPbBr_3$ SC with an applied mask, showing the area of measured mean value.

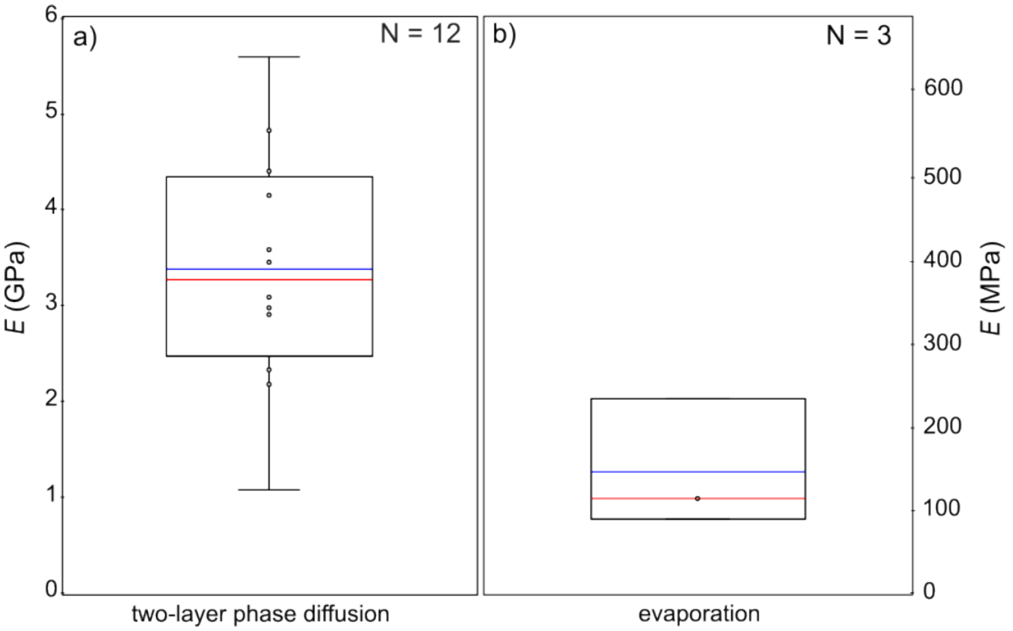

Figure S32: Box plot showing the Young modulus of different SCs by two-layer phase diffusion technique a) and solvent evaporation b). The mean value is indicated by the blue and the median value is indicated by the red line. Sample values are shown as circle points and end-brackets.

References

(1) Maes, J.; Balcaen, L.; Drijvers, E.; Zhao, Q.; De Roo, J.; Vantomme, A.; Vanhaecke, F.; Geiregat, P.; Hens, Z. Light Absorption Coefficient of $CsPbBr_3$ Perovskite Nanocrystals. *J. Phys. Chem. Lett.* **2018**, *9* (11), 3093–3097. https://doi.org/10.1021/acs.jpclett.8b01065.

(2) Ye, J.; Gaur, D.; Mi, C.; Chen, Z.; Fernández, I. L.; Zhao, H.; Dong, Y.; Polavarapu, L.; Hoye, R. L. Z. Strongly-Confined Colloidal Lead-Halide Perovskite Quantum Dots: From Synthesis to Applications. *Chem. Soc. Rev.* **2024**, *53* (16), 8095–8122. https://doi.org/10.1039/D4CS00077C.

(3) Nečas, D.; Klapetek, P. Gwyddion: An Open-Source Software for SPM Data Analysis. *Open Phys.* **2012**, *10* (1), 181–188. https://doi.org/10.2478/s11534-011-0096-2.

(4) Lin, D. C.; Dimitriadis, E. K.; Horkay, F. Robust Strategies for Automated AFM Force Curve Analysis—I. Non-Adhesive Indentation of Soft, Inhomogeneous Materials. *J. Biomech. Eng.* **2007**, *129* (3), 430–440. https://doi.org/10.1115/1.2720924.

(5) Butt, H.-J.; Cappella, B.; Kappl, M. Force Measurements with the Atomic Force Microscope: Technique, Interpretation and Applications. *Surf. Sci. Rep.* **2005**, *59* (1–6), 1–152. https://doi.org/10.1016/j.surfrep.2005.08.003.

(6) Kontomaris, S. V.; Malamou, A. Hertz Model or Oliver & Pharr Analysis? Tutorial Regarding AFM Nanoindentation Experiments on Biological Samples. *Mater. Res. Express* **2020**, *7* (3), 033001. https://doi.org/10.1088/2053-1591/ab79ce.

(7) Slattery, A. D.; Blanch, A. J.; Quinton, J. S.; Gibson, C. T. Accurate Measurement of Atomic Force Microscope Cantilever Deflection Excluding Tip-Surface Contact with Application to Force Calibration. *Ultramicroscopy* **2013**, *131*, 46–55. https://doi.org/10.1016/j.ultramic.2013.03.009.

(8) Miyatani, T.; Fujihira, M. Calibration of Surface Stress Measurements with Atomic Force Microscopy. *J. Appl. Phys.* **1997**, *81* (11), 7099–7115. https://doi.org/10.1063/1.365306.

(9) Sader, J. E.; Chon, J. W. M.; Mulvaney, P. Calibration of Rectangular Atomic Force Microscope Cantilevers. *Rev. Sci. Instrum.* **1999**, *70* (10), 3967–3969. https://doi.org/10.1063/1.1150021.

(10) Giessibl, F. J. Advances in Atomic Force Microscopy. *Rev. Mod. Phys.* **2003**, *75* (3), 949–983. https://doi.org/10.1103/RevModPhys.75.949.

(11) Goodfellow, H. D.; Curd, E. F. Chapter "Physical Fundamentals" in *Industrial Ventilation Design Guidebook*; Elsevier, **2020**; pp 39–109. https://doi.org/10.1016/B978-0-12-816780-9.00004-6.

(12) Houghton, E. L.; Carpenter, P. W.; Collicott, S. H.; Valentine, D. T. Chapter "Basic Concepts and Definitions" in *Aerodynamics for Engineering Students*; Elsevier, **2013**; pp 1–68. https://doi.org/10.1016/B978-0-08-096632-8.00001-1.

(13) Hutter, J. L.; Bechhoefer, J. Calibration of Atomic-Force Microscope Tips. *Rev. Sci. Instrum.* **1993**, *64* (7), 1868–1873. https://doi.org/10.1063/1.1143970.
